# Photoelectrochemical Degradation of Diclofenac, Tetracycline, and Amoxicillin in an Aqueous Sulfate Medium: Analysis of Reactive Species

**DOI:** 10.1021/acsomega.4c10891

**Published:** 2025-02-17

**Authors:** Milda Petruleviciene, Irena Savickaja, Jelena Kovger-Jarosevic, Jurga Juodkazyte, Audrius Padarauskas, Asta Griguceviciene, Arunas Ramanavicius

**Affiliations:** † Department of Chemical Engineering and Technology, Centre for Physical Sciences and Technology, Sauletekio av. 3, LT-10257 Vilnius, Lithuania; ‡ Department of Analytical and Environmental Chemistry, Institute of Chemistry, Faculty of Chemistry and Geosciences, Vilnius University, Naugarduko 24, LT-03225 Vilnius, Lithuania; § Department of Physical Chemistry, Institute of Chemistry, Faculty of Chemistry and Geosciences, Vilnius University, Naugarduko 24, LT-03225 Vilnius, Lithuania; ∥ Department of Nanotechnology, Centre for Physical Sciences and Technology, Sauletekio av. 3, LT-10257 Vilnius, Lithuania

## Abstract

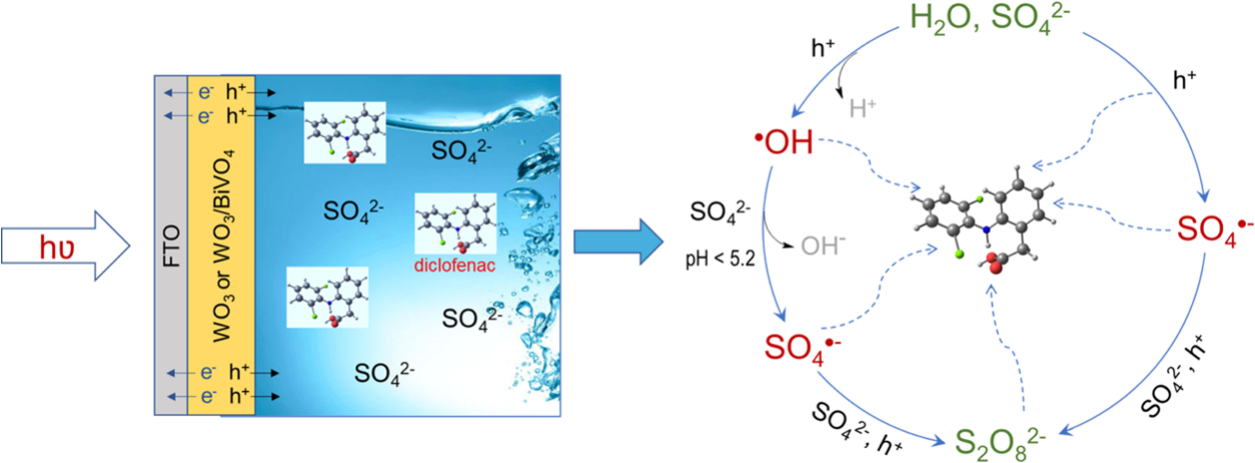

As the environment becomes increasingly polluted, there is a pressing need for the development of effective remediation technologies, particularly in the area of wastewater treatment. Recently, there has been growing interest in advanced oxidation systems (AOSs) based on renewable solar energy. This study focuses on the investigation of photoelectrochemical (PEC) AOSs using WO_3_ and WO_3_/BiVO_4_ photoanodes and an environmentally friendly aqueous sulfate electrolyte for visible light-induced decomposition of pharmaceutical compounds, namely, diclofenac (DCF), amoxicillin (AMX), and tetracycline (TCC). It was demonstrated that in contrast to conventional persulfate-based advanced oxidation processes, where S_2_O_8_^2–^ is activated by UV, ultrasound, or thermal energy to generate highly reactive radical species, in photoelectrochemical systems reported here, radicals were generated by the interaction of photogenerated holes with H_2_O molecules and SO_4_^2–^ ions. These processes eventually led to the formation of S_2_O_8_^2–^ with an estimated Faradaic efficiency of 70–80%. Persulfate has also been shown to contribute to the degradation of pharmaceutical compounds, particularly diclofenac. The degradation efficiencies of AMX, TCC, and DCF were 10–14, 19–21, and 75–80%, respectively, in both PEC–AOSs studied. The formation of the WO_3_/BiVO_4_ heterojunction enhanced charge carrier separation and stability of the photoanode, but the effect on the pharmaceutical decomposition efficiency was not significant. The mechanism of visible light-induced generation of persulfate in the studied PEC systems was analyzed on the basis of thermodynamic considerations and experimental observations of pH variation during photoelectrolysis.

## 1 Introduction

Pharmaceutical compounds detected in aquatic ecosystems have been shown to adversely affect aquatic organisms, raising concerns about the potential contamination of drinking water supplies and highlighting the urgent need to develop innovative technologies to address this issue. Antibiotics, nonsteroidal anti-inflammatory drugs, and other therapeutic agents usually have complex molecular structures that render them resistant to conventional wastewater treatment methodologies.^1−3^ Recently, advanced oxidation processes (AOPs), including UV–H_2_O_2_, Fenton and photo-Fenton, ozone-based technologies, sonolysis, and photocatalysis, have gained significant attention for their effectiveness in degrading pharmaceuticals in aqueous solutions.^4^ Among these, persulfate-based AOPs have emerged as a promising alternative to peroxide-based processes due to their enhanced degradation efficiency, which is attributed to the generation of several different reactive radical species upon persulfate activation.^5−8^ This can be primarily achieved through thermal, photolytic, sonolytic, and radiolytic treatments as well as through reactions with iron oxide magnetic composites, including in situ-formed iron hydroxides and quinones.^9−11^ As a result, AOPs rely on the use of strong oxidants and require significant energy input, prompting the search for more sustainable solutions.

Photoelectrochemical advanced oxidation systems offer the possibility to harness renewable solar energy to induce oxidative degradation of pharmaceutical contaminants.^12,13^ In PEC–AOS, oxidizing species are generated when photoinduced holes in a semiconductor interact with the components of the electrolyte. Therefore, the nature of the semiconductor and the composition of the electrolyte determine which active compounds will be formed.^14,15^ Tungsten oxide (WO_3_) and bismuth vanadate (BiVO_4_) are among the most extensively studied photoanode materials, valued for their moderate band gaps of approximately 2.8 and 2.5 eV, respectively, which enable them to absorb a broad spectrum of visible light.^16,17^ Moreover, they have deep valence band (VB) positions at 3.2 V and 2.6 vs standard hydrogen electrode (SHE), respectively,^18^ thus being able to drive a wide range of oxidation reactions.^19−23^ Pure semiconductors, however, face significant challenges related mainly to the significant recombination of photogenerated electrons and holes and susceptibility to photocorrosion. To address these challenges, various approaches have been investigated, such as forming heterojunctions, incorporating metallic nanoparticles and quantum dots, employing cocatalysts, and adding carbon-based materials.^4^ Among these strategies, heterojunctions composed of two or more directly interfaced semiconductors have proven highly effective in enhancing photocatalytic performance.^24−26^ In a WO_3_/BiVO_4_ heterojunction, charge transfer occurs as a consequence of the alignment of energy levels between the two semiconductors, thereby facilitating the separation of the photogenerated charge carriers. Upon absorbing light, electrons from the valence band (VB) of BiVO_4_ are promoted to the conduction band (CB), creating holes in the VB. Due to the comparatively lower conduction band position of WO_3_, _4_, electrons in the CB of BiVO_4_ can readily transfer to the CB of WO_3_, whereas holes move from VB of In the field of PEC degradation of pharmaceuticals, various heterostructured photoanodes such as Ag-BiVO_4_/BiOI, C_3_N_4_/BiVO_4_, and 20-Cu/TiO_2_ have been tested for the decomposition of diclofenac with reported efficiencies of 68, 68.9, and 71.9%, respectively.^27−29^ The WO_3_/BiVO_4_ heterojunction was applied for degradation of sulfasalazine,^30,31^ norfloxacin,^32^ ibuprofen,^33^ tetracycline,^34,35^ etc. The results summarized in Table 1 show that the PEC degradation of pharmaceuticals is a promising technique. Although many reported studies have been performed using sulfate as the supporting electrolyte, the degradation of pharmaceuticals is typically attributed to the action of photogenerated hydroxyl radicals.^27−29^ However, our previous studies on the PEC activity of WO_3_ and BiVO_4_ photoanodes in an aqueous sulfate medium revealed effective light-assisted formation of persulfate with Faradaic efficiencies (FEs) as high as 80–85%.^14,36^

**Table 1:** Comparison of Pharmaceutical Decomposition Efficiencies Reported in the Literature for Photoelectrochemical and Photocatalytic Advanced Oxidation Systems

Material	Pharmaceutical compound	Decomposition efficiency (%)	Electrolyte	Concentration of pharmaceutical compound	Reference
Co_3_O_4_/WO_3_ (photocatalysis)	diclofenac	98.7		15 ppm	(37)
WO_3_/Fe_2_O_3_	diclofenac	40	H_2_SO_4_ (pH 2)	10 mg L^–1^	(38)
Ag-BiVO_4_/BiOI	diclofenac	92	0.1 M Na_2_SO_4_	10 mg L^–1^	(27)
ZnO-WO_3_ (photocatalysis)	diclofenac	76		10–25 mg L^–1^	(39)
N, S–TiO_2_/TiO_2_ NTs	diclofenac	73.3		5 mg L^–1^	(40)
TiO_2_ NPs/TiO_2_ NTAs	diclofenac	63.6	0.1 M Na_2_SO_4_	5 mg L^–1^	(41)
2D-on-2D WS_2_@CoFe_2_O_4_	amoxicillin	99	1 M KOH	10 mg L^–1^	(42)
Ba(Ti_0.9_Sc_0.05_Nb_0.05_)O_3_ (BTSN) (photocatalysis)	amoxicillin	92	alkaline (pH 11)	50 mg L^–1^	(43)
WO_3_ nanoplates	tetracycline	72	0.5 M Na_2_SO_4_	5 mg L^–1^	(4)
g-C_3_N_4_ nanosheets/TiO_2_ nanotube	tetracycline	93	0.1 M Na_2_SO_4_	10 mg L^–1^	(4)
BiVO_4_/ZnO	tetracycline	66.1	0.1 M Na_2_SO_4_	20 mg L^–1^	(4)
WO_3_/Au/FeOOH	tetracycline	98	0.1 M Na_2_SO_4_ (pH 6.2)	20 mg L^-1^	(34) and (44)
WO_3_/BiVO_4_	tetracycline	91	0.1 M Na_2_SO_4_	10 mg L^–1^	(34) and (35)
WO_3_/BiVO_4_ (photocatalysis)	sulfasalazine	65–90		9 mg L^–1^	(30)
WO_3_/BiVO_4_	sulfamethoxazole	35–75	NaCl	25 mg L^–1^	(31)
WO_3_/BiVO_4_	norfloxacin	70	0.1 M Na_2_SO_4_	10 mg L^–1^	(32)

The objective of this study was to elucidate the impact of in situ-generated persulfate on the degradation of pharmaceutical compounds, specifically diclofenac , amoxicillin, and tetracycline, in PEC–AOSs with WO_3_ or WO_3_/BiVO_4_ photoanodes and a simple, environmentally friendly sulfate electrolyte. From the standpoint of applicability, this approach is considerably more straightforward and cost-effective, as it removes the need for costly persulfates and UV illumination. Furthermore, the method allows for the utilization of sulfate ions, which are frequently present in various wastewaters.

Hydrothermal and sol–gel synthesis methods were used to form semiconductor photoelectrodes. Materials were characterized using X-ray diffraction (XRD), scanning electron microscopy (SEM), and energy-dispersive X-ray (EDX) analysis techniques. Photoelectrochemical properties were evaluated using cyclic voltammetry (CV), chronoamperometry (CA), and electrochemical impedance spectroscopy (EIS). The efficiency of light-driven decomposition of DCF, AMX, and TCC was evaluated using UV–vis spectrometry, and the mechanisms of light-induced reactive species formation were analyzed using radical quenching with scavengers. The study demonstrated the significant influence of local pH on the complex interactions occurring during the light-induced formation of reactive radical species. Furthermore, the crucial role of the nature of the semiconductor, which is in direct contact with the electrolyte, on the degradation efficiency was revealed.

## 2 Experimental Section

### 2.1 Materials

Bismuth (III) nitrate pentahydrate (Bi(NO_3_)_3_·5H_2_O, 98.0%) from Thermo Fisher Scientific (Waltham, Massachusetts, USA), vanadyl acetylacetonate (C_10_H_14_O_5_V, 99%) from Acros Organics (Geel, Belgium), acetic acid glacial (CH_3_COOH, 99.9%) from Reachem (Bratislava, Slovakia), acetylacetone (C_5_H_8_O_2_, 99.0%) from Chempur (Piekary Slaskie, Poland), hydrochloric acid (HCl, 35–38%) from Stanlab (Lublin, Poland), sodium sulfate anhydrous (Na_2_SO_4_, 99.5%) from Chempur (Piekary Slaskie, Poland), methanol (CH_3_OH, 99,8%) from Chempur (Piekary Slaskie, Poland), *tert*-butanol ((CH_3_)_3_COH, 99.5%) from Thermo Scientific (Waltham, Massachusetts, USA), sodium tungstate dihydrate (Na_2_WO_4_·2H_2_O, 99.0%) from Carl Roth (Karlsruhe, Germany), ammonium oxalate monohydrate ((NH_4_)_2_C_2_O_4_·H_2_O, 99.0%) from Chempur (Piekary Slaskie, Poland), poly(ethylene glycol) (PEG (C_2_H_4_O)*_n_*H_2_O) from Carl Roth (Karlsruhe, Germany), diclofenac sodium salt (C_14_H_10_C_l2_NNaO_2_, ≥98.0%) from Farmalabor (Canosa Di Puglia, Italia), tetracycline hydrochloride (C_22_H_24_N_2_O_8_·HCl, ≥95.0%), amoxicillin (C_16_H_19_N_3_O_5_S, 95.0–102.0%) from Sigma-Aldrich (Burlington, Massachusetts, USA), sodium borate decahydrate (Na_2_B_4_O_7_·10H_2_O, ≥95.0%) from Tarchem, Tornowskie Gory, Poland), and boric acid (H_3_BO_3_, ≥95.0%) from Chempur (Piekary Slaskie, Poland) were used as received from suppliers without further purification.

### 2.2 Synthesis of WO_3_ and WO_3_/BiVO_4_ Coatings

WO_3_ coatings on fluorine-doped tin oxide (FTO) substrates were synthesized using the hydrothermal synthesis procedure.^36^ First, 0.2593 g of Na_2_WO_4_·2H_2_O was dissolved in 30 mL of deionized water. After that, 6 mL of 3 M HCl was added and the solution was stirred for 10 min with a magnetic stirrer at room temperature (∼20 °C). Then, 0.2 g of (NH_4_)_2_C_2_O_4_·2H_2_O and PEG in a molar ratio of W:PEG equal to 1:2 were added to the solution as complexing and structure directing agents, respectively, and the mixture was stirred for 20 min. Next, 34 mL of deionized water were added, and the solution was stirred for 5 min. The resulting mixture was transferred to a stainless steel autoclave. FTO substrates, precleaned in acetone, isopropanol, and water under ultrasonication, were then immersed in the face-down position, and the synthesis was performed at 160 °C for 24 h. Afterwards, the autoclave was left to cool to room temperature. The synthesized coatings were washed 3 times with deionized water and dried in air at 60 °C for 4 h.

A WO_3_/BiVO_4_ heterostructure was formed by depositing a BiVO_4_ layer on top of a previously synthesized WO_3_ coating. BiVO_4_ sol–gel was prepared by mixing 0.9 mL of 0.2 M Bi(NO_3_)_3_·5H_2_O solution in glacial acetic acid and 6 mL of 0.03 M C_10_H_14_O_5_V solution in acetylacetone. Acetic acid was used for pH control and as a chelating agent, which stabilized Bi in the solution by preventing hydrolysis and precipitation of Bi(OH)_3_. Acetylacetonate played the role of a chelating agent for the vanadium precursor. The resulting sol–gel was green in color. WO_3_-coated FTO slides were immersed into the sol–gel and kept there for 24 h. Finally, the WO_3_/BiVO_4_ coatings were annealed at 400 °C for 2 h in an air atmosphere with a heating ramp of 1 °C min^–1^. During annealing, the byproducts of the reaction were released in the form of volatile organic compounds and gases (NO_*x*_, CO_2_). For some comparative measurements, a BiVO_4_ coating was deposited directly on the FTO substrate.

### 2.3 Characterization of the Structure, Surface Morphology, and Chemical Composition of WO_3_ and WO_3_/BiVO_4_ Coatings

The crystalline structures of synthesized WO_3_ and WO_3_/BiVO_4_ coatings were investigated using an X-ray diffractometer SmartLab (Rigaku) equipped with a 9 kW rotating Cu anode X-ray tube. The analysis covered a 2θ range of 20–80°, utilizing the grazing incidence X-ray diffraction (GIXRD) method with a 0.5° angle (ω) set between the parallel beam of X-rays and the specimen surface. Phase identification was conducted using Match software and the crystallography open database (COD).

WO_3_ crystallite size in pure and heterostructured coatings was evaluated according to the following Scherrer equation
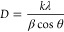
1where *D* is the crystallite size in nm, *k* is the Scherrer constant equal to 0.9, *λ* is the X-ray wavelength equal to 0.15406 nm, *β* is the full width at half-maximum of the diffraction peak (in radians), and *θ* is Bragg’s angle in radians. The three peaks at 2*θ* positions of 23.6, 24.4, and 34.2° were chosen for the evaluation of *D*.

The surface morphology and chemical composition of the prepared samples were analyzed using a Helios NanoLab dual beam workstation (Oxford Instruments, the Netherlands) equipped with an energy-dispersive X-ray (EDX) spectrometer at 10 and 20 kV acceleration voltage.

The X-ray photoelectron spectroscopy (XPS) analyses were carried out with a Kratos Axis Supra spectrometer using a monochromatic Al K(α) source (25 mA, 15 kV). The instrument work function was calibrated to give a binding energy (BE) of 83.96 eV for the Au 4f_7/2_ line for metallic gold, and the spectrometer dispersion was adjusted to give a BE of 932.62 eV for the Cu 2p_3/2_ line of metallic copper. The Kratos charge neutralizer system was used on all specimens. Survey (wide) scan analyses were carried out with an analysis area of 300 μm × 700 μm and a pass energy of 160 eV. High-resolution analyses were carried out with an analysis area of 300 μm × 700 μm and a pass energy of 20 eV. Spectra were charge-corrected to the main line of the carbon 1s spectrum (adventitious carbon) set to 284.8 eV. Spectra were analyzed using CasaXPS software (version 2.3.23rev1.1R).

### 2.4 Photoelectrochemical Investigations

Cyclic voltammetry, chronoamperometry, and electrochemical impedance spectroscopy measurements were performed using a potentiostat/galvanostat Zennium/Zahner Xpot (Zahner Elektrik, Germany), a three-electrode electrochemical cell, and 0.1 M Na_2_SO_4_ electrolyte. WO_3_ and WO_3_/BiVO_4_ coatings on the FTO substrates were used as working electrodes. A silver/silver chloride electrode with saturated KCl solution (Ag/AgCl_(sat. KCl)_) and a Pt plate (1  ×  1 cm^2^) were used as reference and counter electrodes, respectively. All potential values in the paper are reported vs Ag/AgCl_(sat. KCl)_ unless noted otherwise. The electrodes were illuminated from the back side using an light-emitting diode (LED) solar simulator (Redoxme AB, Sweden), which provides illumination approximating natural sunlight (AM1.5G) in the wavelength range of 400–1100 nm with an intensity of 100 ± 2 mW cm^–2^. EIS measurements were carried out at 0.7 V with an AC voltage amplitude of ±10 mV, within a frequency range from 10^4^ to 0.1 Hz under illumination.

Applied bias photon-to-current efficiency (ABPE) measurements were performed in a two-electrode setup with WO_3_ or WO_3_/BiVO_4_ photoanodes and a Pt wire cathode (Figure S1). The photocurrent was recorded in a solution of 0.1 M Na_2_SO_4_, with an applied voltage ranging from 0 to 2.5 V (vs Pt) and a potential scan rate of 50 mV s^–1^. ABPE was calculated according to the following equation:^45,46^
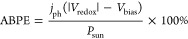
2where *j*_ph_ is the photocurrent density (mA cm^–2^), *V*_redox_ is the thermodynamic potential of a reaction (V), *V*_bias_ is the applied bias (V), and *P*_sun_ is the illumination power density (100 mW cm^–2^). *V*_redox_ was evaluated, presuming that the photoanodic reaction in Na_2_SO_4_ electrolyte was oxidation of SO_4_^2–^ to S_2_O_8_^2–^ and the cathodic one was the hydrogen evolution reaction. Given that standard potentials *E*_a_ and *E*_c_ of the anodic and cathodic half-reactions, respectively, are^36,47^
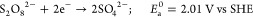
3and
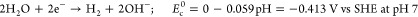
4

the thermodynamic potential of the cell reaction is calculated as *V*_redox_ = *E*_c_^0^ – *E*_a_^0^ = −0.413 – 2.01 = −2.423 V. A negative *V*_redox_ indicates that the SO_4_^2–^ oxidation reaction (eq 5) is not a spontaneous process.
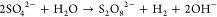
5

The formation of reactive sulfate species (RSS) was investigated via photoelectrolysis in a 0.1 M Na_2_SO_4_ electrolyte in a two-electrode cell at an applied voltage of 1.4 V (vs Pt) until a total charge, *Q*, of approximately 0.5 C was passed through the system. The electrolyte from the anodic compartment of the cell was then collected and analyzed for the presence of RSS in the form of S_2_O_8_^2–^ using chromatometric titration. The Faradaic efficiency (FE, %) of the photoelectrochemical generation of persulfate was calculated as the ratio *m*_exp_/*m*_theor_ × 100, where *m*_exp_ is the experimentally measured mass of S_2_O_8_^2–^ and *m*_theor_ is the theoretical mass determined using Faraday’s law based on the electric charge passed through the cell during photoelectrolysis. The calculation assumes that the only anodic reaction occurring on the photoanode is the two-electron oxidation of SO_4_^2–^ into S_2_O_8_^2–^. Detailed titration protocols for determining persulfate are reported in our previous studies.^14,48^

Variation of electrolyte pH during photoelectrolysis was measured using a pH meter FiveEasy Plus FP20 (Mettler Toledo, USA).

### 2.5 Investigation of Decomposition of Pharmaceutical Compounds

Studies on photoelectrochemical decomposition of pharmaceutical compounds were performed in a two-electrode cell with 0.1 M Na_2_SO_4_ electrolyte containing 50 mg L^–1^ of DCF, AMX, or TCC at an applied voltage of 1.4 V (vs Pt). 0.1, 0.3, 0.5, and 1 C of charge were passed through the cell in separate experiments. A fresh portion of the electrolyte was used each time. After photoelectrolysis, the solution from the anodic compartment of the cell was collected, and the degradation efficiency, *η*, was evaluated using a UV–vis spectrophotometer Lambda 35UV/VIS (PerkinElmer, USA)
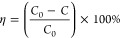
6where *C*_0_ is the initial concentration and *C* is the remaining concentration of a pharmaceutical.

To compare the decomposition efficiency under different conditions, solutions of DCF, AMX, and TCC were also subjected to photocatalytic, electrochemical, and chemical oxidation treatments. In the latter case, K_2_S_2_O_8_ was added to 0.1 M Na_2_SO_4_ containing 50 mg L^–1^ of DCF, AMX, or TCC. The calculated amounts of the oxidant corresponded to the mass of persulfate that would be formed during photoelectrolysis after passing 0.1, 0.3, 0.5, or 1 C of charge, assuming that the FE of the PEC persulfate generation was 100%. After the addition of the oxidant, the electrolytes were kept for 25 h to provide enough time for the reaction to proceed, and after that, the efficiency of pharmaceutical decomposition was evaluated spectrophotometrically (eq 6). In photocatalytic treatment, no external potential was applied, and the system was illuminated for the same periods of time, which were required in the PEC experiment to pass 0.1, 0.3, 0.5, or 1 C of charge. Similarly, in electrochemical oxidation experiments, electrolysis at 1.4 V (vs Pt) in the dark was performed for the same periods of time. The electrolytes were then collected and subjected to spectrophotometric analysis.

### 2.6 Studies of the DCF Decomposition Mechanism

The mechanism of photoelectrochemical decomposition of DCF was studied using *tert*-butanol (*t*-BuOH), methanol (MeOH), and ammonium oxalate (AO) as scavengers for sulfate radicals, hydroxyl radicals, and photogenerated holes, respectively. Experiments were performed with 0.1 M Na_2_SO_4_ electrolyte containing 50 mg L^–1^ of DCF and 2 mM of scavenger. Three different electrolytes were prepared for this purpose. The parameters of the PEC–AOS system were the same as those in the photoelectrochemical experiments described above. Samples of the electrolyte for the spectrophotometric evaluation of DCF concentration were sequentially collected from the anodic compartment of the cell after passage of 0.1, 0.3, 0.5, and 1 C of charge.

## 3 Results and Discussion

### 3.1 Structural and Morphological Analysis of the Coatings

**Figure 1:**
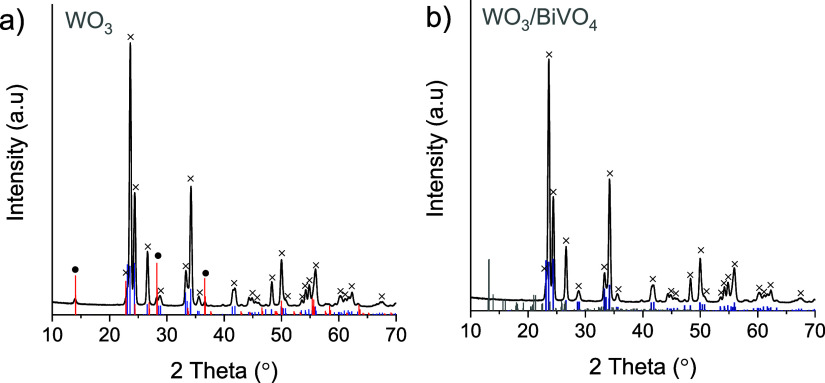
XRD spectra of WO_3_ (a) and WO_3_/BiVO_4_ (b) coatings (×—monoclinic WO_3_; •—hexagonal WO_3_). Blue, red, and gray columns correspond to reference spectra of monoclinic WO_3_ (COD: 2106383), hexagonal WO_3_ (COD: 1004058), and monoclinic BiVO_4_ (COD: 9013437), respectively.

X-ray diffraction analysis was conducted to assess the crystalline structure of the synthesized coatings. Peaks corresponding to the monoclinic (COD: No. 2106382) and hexagonal (COD: 1004057) phases can be observed in the XRD spectra of WO_3_ (Figure 1a). The diffractogram of the WO_3_/BiVO_4_ sample displays only the peaks of monoclinic WO_3_ (Figure 1b). The absence of the hexagonal phase in the latter case can be explained by its transformation into monoclinic during the additional annealing applied in the synthesis of WO_3_/BiVO_4_, since the hexagonal structure is metastable and is usually formed at lower annealing temperatures.^49^ This additional annealing, however, did not affect the WO_3_ crystallite size, which was found to be 25.3 nm for both WO_3_ and WO_3_/BiVO_4_. Peaks attributable to the BiVO_4_ crystalline phase are absent in the diffractograms of the WO_3_/BiVO_4_ heterostructure (Figure 1b), indicating that the bismuth vanadate layer is extremely thin (Figure S2).

**Figure 2:**
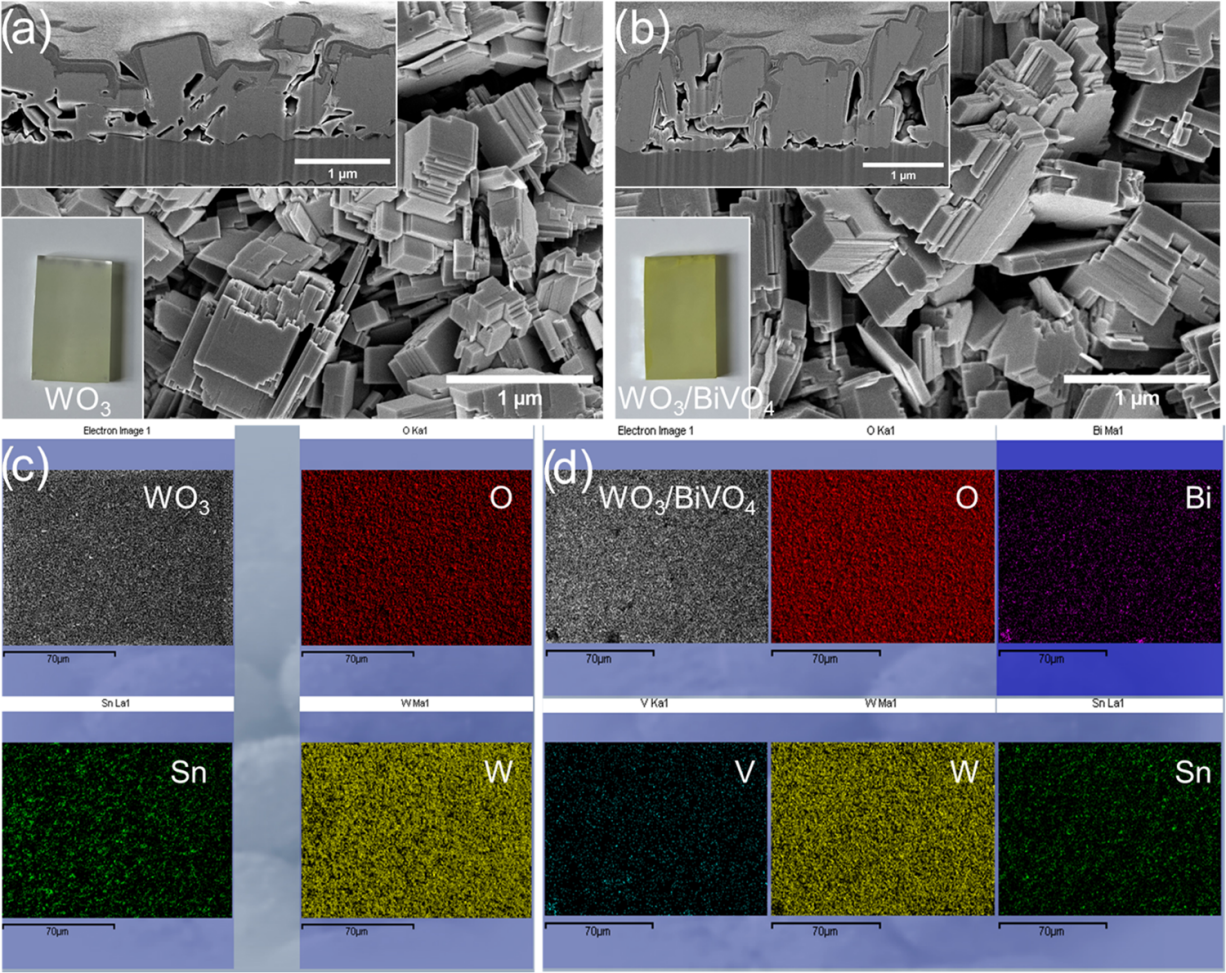
SEM (a, b) and EDX elemental mapping (c, d) images of WO_3_ and WO_3_/BiVO_4_ coatings.

The surface morphologies of the layers were also found to be very similar. The coatings consisted of large cubic WO_3_ crystals, up to one micron in size, with stepped edges (Figure 2a,b). Although BiVO_4_ is scarcely discernible in SEM images, the lower insets in Figure 2a,b clearly demonstrate the different colors of WO_3_ and WO_3_/BiVO_4_ samples. To confirm the deposition of BiVO_4_, energy-dispersive X-ray spectroscopy and X-ray photoelectron spectroscopy analyses were performed. The EDX results presented in Table 2 and Figure S2, in conjunction with the elemental mapping images in Figure 2c,d, demonstrate the presence of Bi and V elements in the heterostructured sample. The chemical states of the elements were subsequently verified through XPS.

**Table 2:** EDX Analysis Results for the WO_3_ and WO_3_/BiVO_4_ Samples

	Element (atom %)				
Sample	O	W	Sn	V	Bi
WO_3_	71.25	80.33	1.98		
WO_3_/BiVO_4_	70.02	26.76	2.03	0.72	0.48

High-resolution spectra of both WO_3_ and WO_3_/BiVO_4_ (Figure S3a–d) exhibit peaks at 35.7 and 37.85 eV corresponding to the W 4f_7/2_ and W 4f_5/2_ electrons in the W^6+^ oxidation state, whereas the peak at 530.4 eV is attributed to the O 1s electron in O^2–^.^33,50^ Peaks at 159.5 and 169.8 eV in the spectrum of the heterostructured sample are assigned to Bi 4f_7/2_ and Bi 4f_5/2_ electrons in Bi^3+^, respectively (Figure S3e), and peaks at 517 and 524 eV correspond to V 2p_3/2_ and V 2p_1/2_ electrons of V^5+^, respectively (Figure S3f).^50−52^ Small features attributable to V^4+^ can be seen in the lower binding energy range (Figure S3f). The Bi/V ratio was found to be ∼1:1, while the presence of W peaks in the spectra of the WO_3_/BiVO_4_ surface suggests that the BiVO_4_ layer may not form continuous coverage over the WO_3_ surface.

### 3.2 Photoelectrochemical Analysis

**Figure 3:**
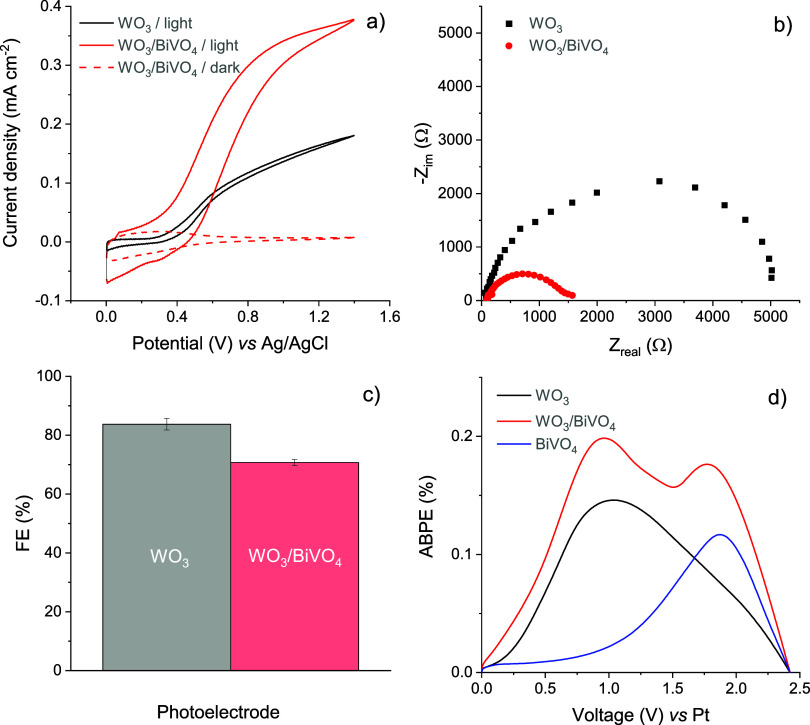
Characterization of the photoelectrochemical activity of WO_3_ and WO_3_/BiVO_4_ coatings in 0.1 M Na_2_SO_4_ electrolyte: (a) cyclic voltammograms recorded at 50 mV s^–1^ scan rate, (b) Nyquist plots recorded at 0.7 V, (c) Faradaic efficiencies of PEC generation of persulfate, and (d) applied bias photon-to-current efficiencies of WO_3_, BiVO_4_, and WO_3_/BiVO_4_ photoelectrodes.

The cyclic voltammetry was employed for the preliminary assessment of the PEC activity of the synthesized WO_3_ and WO_3_/BiVO_4_ coatings in 0.1 M Na_2_SO_4_ electrolyte (Figure 3a). In the absence of illumination, the current was negligible, whereas under illumination, the maximum photocurrents, *j*_ph_, generated by WO_3_ and WO_3_/BiVO_4_ were 0.18 and 0.38 mA cm^–2^, respectively. The fact that the photocurrent of the WO_3_/BiVO_4_photoelectrode was almost twice as high as that of WO_3_ and the onset of *j*_ph_ was observed at lower potentials, clearly indicates that the formation of a heterojunction significantly facilitates the separation and transfer of photogenerated charge carriers. This was corroborated by electrochemical impedance spectroscopy measurements, which showed that charge transfer resistance, *R*_ct_, proportional to the diameter of the semicircle in the Nyquist plot, was more than 3 times larger for WO_3_ electrodes compared to WO_3_/BiVO_4_ (Figure 3b). The imaginary impedance, which corresponds to the electrochemical capacitance of the coating, was higher for the WO_3_/BiVO_4_ heterojunction compared to the pure WO_3_ coating.

The analysis of the products of photoanodic processes occurring on the surface of WO_3_ and WO_3_/BiVO_4_ electrodes in 0.1 M Na_2_SO_4_ revealed the predominant formation of reactive sulfate species. The FE of PEC generation of S_2_O_8_^2–^ on WO_3_ was approximately 82%, whereas for WO_3_/BiVO_4_, it was around 70% (Figure 3c). The slight decrease in FE due to the deposition of the BiVO_4_ layer on WO_3_ is most likely an indication of a more significant contribution of the competing light-induced oxygen evolution reaction (OER), which is the most probable competing photoanodic process in the studied system.^4,8,53^ A sample of pure BiVO_4_ on FTO was also prepared and tested for comparison. However, the photocurrent of a thin BiVO_4_ layer was found to be very low, measuring several microamperes. Furthermore, no photoelectrochemically generated persulfate was found by the titrimetric analysis of the electrolyte after more than 2 h of photoelectrolysis or the amount of S_2_O_8_^2–^ was below the detection limit of the analytical method used. Further analysis was conducted to evaluate the applied bias photon-to-current efficiency of reaction 5 in the studied system. As shown in Figure 3d, the ABPE plot of the WO_3_/BiVO_4_ sample revealed the presence of two peaks at positions corresponding to those of the pure WO_3_ and BiVO_4_ materials. However, the values of ABPE for the heterostructure were higher, suggesting the enhancement of electronic properties in the composite. A similar phenomenon was reported in ref (54), where double peaks in incident photon-to-current efficiency (IPCE) plots were observed for the WO_3_/BiVO_4_ heterojunction.

### 3.3 Study of Light-Induced Decomposition of Diclofenac, Tetracycline, and Amoxicillin in an Aqueous Sulfate Medium

**Figure 4:**
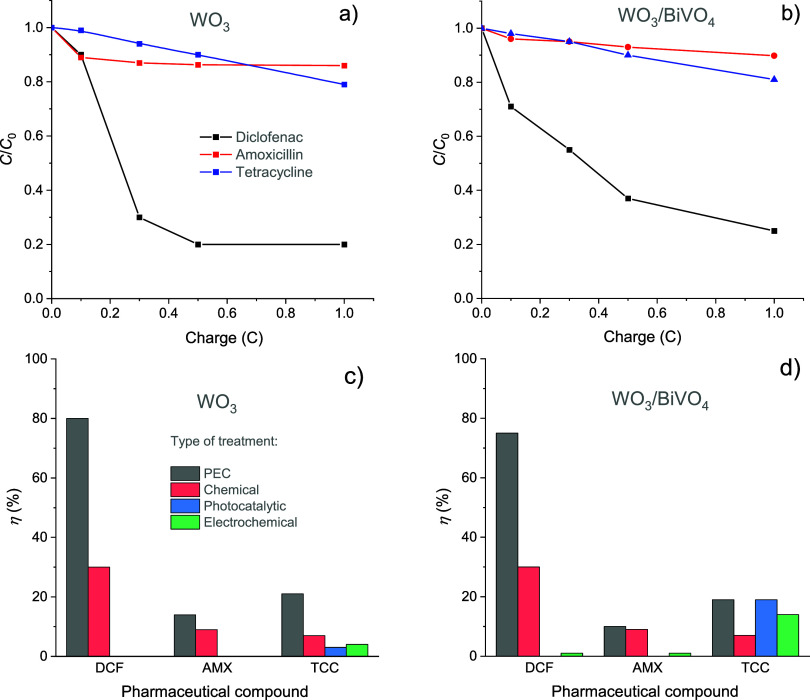
Progression of pharmaceutical compound degradation depicted by the normalized concentration ratio (*C*/*C*_0_) as a function of charge consumed during photoelectrolysis of 0.1 M Na_2_SO_4_ solution containing 50 mg L^–1^ of DCF, AMX, or TCC using (a) WO_3_ or (b) WO_3_/BiVO_4_ photoelectrode. Comparison of pharmaceutical compound degradation efficiency under different oxidative treatments including chemical with S_2_O_8_^2–^, photoelectrochemical, electrochemical, and photocatalytic treatments in AOS systems with (c) WO_3_ and (d) WO_3_/BiVO_4_ (see the Section 2 for the detailed description of conditions applied in these experiments).

WO_3_ and WO_3_/BiVO_4_ samples were further used in the studies of light-assisted degradation of biologically active compounds in an aqueous sulfate medium. A solution of 0.1 M Na_2_SO_4_ containing 50 mg L^–1^ of diclofenac, amoxicillin, or tetracycline was subjected to photoelectrolysis, as described in the Section 2. Decomposition efficiencies as a function of charge consumed in photoelectrolysis are compared in Figure 4. It is worth noting that the normalized concentration ratio (*C*/*C*_0_) is typically plotted against time rather than the charge. However, due to fluctuations in photocurrent, plotting it against charge was deemed more appropriate and accurate. The degradation of DCF was clearly the most effective among the pharmaceutical compounds studied and occurred significantly faster on WO_3_ than on WO_3_/BiVO_4_. For WO_3_, approximately 80% (±5%) decomposition was achieved after the passage of just 0.5 C, whereas for WO_3_/BiVO_4_, a charge of 1 C was required to achieve a similar level of DCF decomposition. The PEC degradation of AMX and TCC occurred at a markedly slower rate and with a significantly lower efficiency for both photoelectrodes. Further experiments were conducted to observe the progression of pharmaceutical degradation over time in electrolytes that underwent PEC treatment. For this purpose, the collected samples were subjected to repeated UV–vis spectroscopic analysis after a period of 48 h. Figure S4 illustrates that in the case of DCF, almost complete decomposition (*η* = 96 ± 5%) was achieved with both photoanodes, whereas for AMX and TCC, the degradation progress was slight to negligible.

These findings illustrate that despite the enhanced charge separation in the WO_3_/BiVO_4_ heterojunction, the decomposition efficiency of the studied compounds is strongly influenced by the intrinsic properties of the semiconductor, which is in direct contact with the electrolyte, as well as the chemical composition of pharmaceuticals themselves.^34,55,56^ Therefore, it is important to optimize the PEC–AOSs to achieve complete decomposition of studied antibiotics.^34,57,58^

The efficiency of pharmaceutical degradation in the PEC–AOS system was compared with the results obtained when these compounds were subjected to either electrochemical, photocatalytic, or chemical oxidative treatments. In the latter case, the calculated amount of S_2_O_8_^2–^, corresponding to *Q* of 0.1, 0.3, 0.5, or 1 C and assuming the Faradaic efficiency of SO_4_^2–^ oxidation to S_2_O_8_^2–^ of 100%, was added to 0.1 M Na_2_SO_4_ electrolyte containing 50 mg L^–1^ of DCF, TCC, or AMX. The photoelectrochemical treatment demonstrated superior efficiency in the decomposition of DCF when compared to all other treatments involving both photoelectrodes (Figures 4c,d and S4). The average *η* for DCF was ∼80%, whereas for AMX, it ranged between 10 and 14% and remained at approximately 20% for TCC. Another obvious fact is that photocatalytic and electrochemical treatments were absolutely ineffective in the degradation of DCF and AMX for both WO_3_ and WO_3_/BiVO_4_. In the case of TCC, different results were obtained with the WO_3_/BiVO_4_ heterojunction, where degradation efficiencies of 20 and 17% were achieved for photocatalytic and electrochemical treatments, respectively (Figure 4d). The chemical treatment with S_2_O_8_^2–^ was found to be more efficient for the degradation of DCF than that of the other two compounds. However, *η* was only 30%, which is almost three times lower than that of the PEC treatment with any of the photoelectrodes. The chemical action of the photoelectrochemically generated S_2_O_8_^2–^ (Figure 3c) can explain the progression of DCF degradation over the 48 h period after the PEC treatment described above (Figure S4). Nevertheless, these findings suggest that in addition to S_2_O_8_^2–^, some other reactive species may also be involved in the degradation of the assessed pharmaceuticals. Given that the PEC degradation of diclofenac was the most facile, further studies were conducted to gain deeper insights into the mechanism of light-assisted decomposition of this compound and the nature of the reactive species.

### 3.4 Investigation of the Diclofenac Decomposition Mechanism

Radical species that may be generated by the interaction of photoinduced holes with the components of the Na_2_SO_4_ electrolyte are as follows^47^
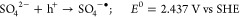
7
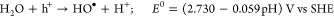
8

It is noteworthy that the potential of hydroxyl radical formation (eq 8) as well as the potentials of conduction and valence band (CB and VB) of metal oxide semiconductors are pH dependent and shift negatively by 0.059 pH with increasing pH.^59,60^ Considering that the VB positions in WO_3_ and BiVO_4_ at pH = 0 are ∼3.2 eV^60^ and ∼2.9 V,^59^ respectively, it can be inferred that photogenerated holes would be sufficiently energetic to oxidize electrolyte species to highly reactive HO^•^ and SO_4_^–•^ radicals. The formation of these radicals has been evidenced in numerous studies using such techniques as electron paramagnetic resonance, transient absorption spectroscopy, quenching, etc.^8,53,54^ The latter approach was applied in this study to investigate the mechanism of the photoelectrochemical degradation of DFC with WO_3_ and WO_3_/BiVO_4_ photoanodes.

Ammonium oxalate (AO), *tert*-butyl alcohol (TBA), or methanol (MeOH) was added to 0.1 M Na_2_SO_4_ + 50 mg L^–1^ DCF electrolyte as h^+^ or radical (HO^•^, SO_4_^–•^) scavengers^61−63^ and PEC degradation of DCF was monitored as previously described. According to the literature, TBA is used as the HO^•^ scavenger,^63,64^ and MeOH as the SO_4_^–•^ scavenger;^63^ however, in fact, both alcohols scavenge both radicals, but at significantly different rates.^65^ As shown in Table 3, the reaction of MeOH with HO^•^ is about 300 times faster than that with SO_4_^–•^, while in the case of TBA, this difference is even larger, i.e., from 400 to 1900. Another important aspect is that MeOH reacts faster with both radicals compared to TBA.

**Table 3:** Radical Scavenging Reaction Rate Constants^a^

	Reaction rate constant (M^–1^ s^–1^)	
Scavenger	SO_4_^–•^	HO^•^
MeOH	3.2 × 10^6^	9.7 × 10^8^
TBA	(4–9.1) × 10^5^	(3.8–7.6) × 10^8^

a Adapted with permission from ref (65). Copyright 2009 American Chemical Society.

**Figure 5:**
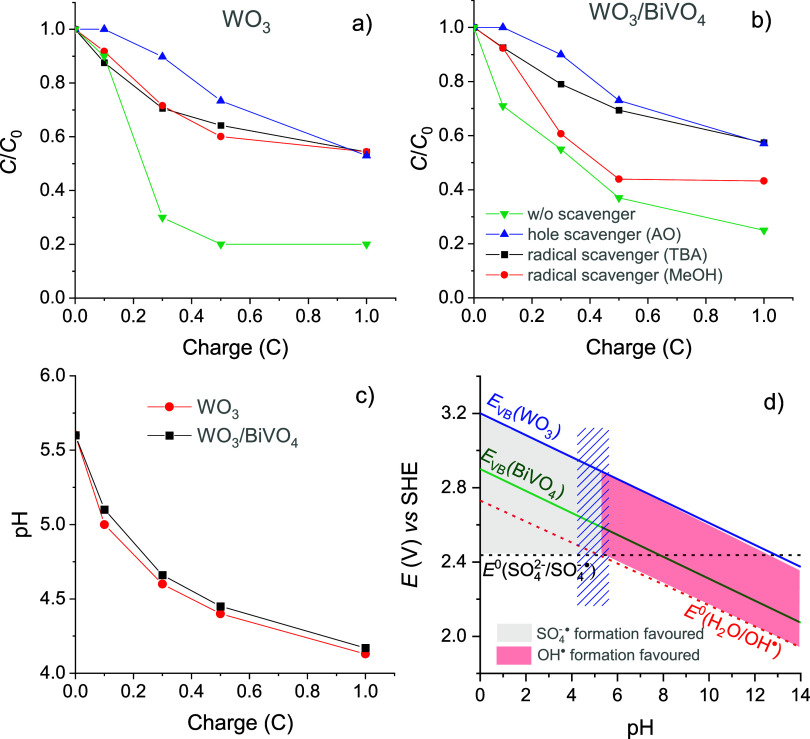
Progression of DCF degradation as a function of charge consumed in the photoelectrolysis of 0.1 M Na_2_SO_4_ + 50 mg L^–1^ DFC solution containing 2 mM of AO, TBA, or MeOH as h^+^, HO^•^, or SO_4_^–•^ scavengers, respectively, using (a) WO_3_ or (b) WO_3_/BiVO_4_ photoelectrode. (c) Variation of electrolyte pH during photoelectrolysis. (d) pH dependence of the energy levels of h^+^ in WO_3_ and BiVO_4_ as well as H_2_O/HO^•^ and SO_4_^2–^/SO_4_^–•^ redox couples; gray and pink shaded areas represent the pH ranges where, respectively, the formation of either SO_4_^–•^ or HO^•^ by photoinduced holes is thermodynamically favored; blue patterned area shows the working pH range.

The results presented in Figure 5a,b demonstrate that the quenching of holes with OA had the most pronounced effect in suppressing the degradation of DFC for both photoanodes. However, the efficacy of AO diminished over time, likely due to the consumption of this scavenger during longer experiments. In the case of WO_3_ (Figure 5a), the addition of TBA or MeOH had almost the same effect on the DFC degradation rate, with more than 50% of DCF remaining undecomposed when a charge equal to 1 C was passed through the electrochemical cell. Considering the significant influence of electrolyte pH on the arrangement of energetic levels of HO^•^ and SO_4_^–•^ formation, variation of 0.1 M Na_2_SO_4_ solution pH during photoelectrolysis with both photoelectrodes was evaluated in a separate experiment (Figure 5c). After the passage of 1 C of charge, the pH of the electrolyte decreased from an initial value of 5.6 to 4.2 . Slightly acidic initial pH of 0.1 M Na_2_SO_4_ solution can be explained by the absorption of CO_2_ from the air. In order to understand how the changes in the electrolyte pH affect the arrangement of energy levels of the reactant species, specifically the holes in the VB of WO_3_ and BiVO_4_, with respect to the potentials of HO^•^/H_2_O and SO_4_^–•^/SO_4_^2–^, the *E*–pH diagram was plotted and is shown in Figure 5d. It can be seen that at a pH of ∼5.2, the potentials of the reactions 7 and 8 become practically equal. Consequently, at pH > 5.2, the formation of HO^•^ is thermodynamically favored, whereas at pH < 5.2, the formation of SO_4_^–•^ becomes more favorable. Another important aspect to consider is that at pH < 5.2, HO^•^ can oxidize SO_4_^2–^ to SO_4_^–•^, whereas at pH > 5.2, SO_4_^–•^ can oxidize H_2_O to HO^•^. The working pH range (Figure 5c) is just around 5.2, which means that the potentials of HO^•^ and SO_4_^–•^ formation are rather close in both photoelectrochemical systems studied. The decrease in pH was a little faster in the case of the WO_3_ photoelectrode, implying that the probability of SO_4_^–•^ formation was increasing faster. This is in agreement with slightly higher FEs of S_2_O_8_^2–^ formation observed for the WO_3_ photoelectrode (Figure 3c). In the case of the WO_3_/BiVO_4_ photoelectrode, the influence of the scavengers on the degradation of DCF was different: the presence of TBA suppressed the degradation rate of diclofenac more significantly than the addition of MeOH (Figure 5b). Although the effects of both scavengers were almost equal up to *Q* = 0.1 C, the influence of MeOH was inferior at longer photoelectrolysis times. It is difficult to explain this phenomenon, especially since the quenching of HO^•^ with TBA had a very strong effect on DCF degradation, and MeOH should quench these radicals even faster. Such a result could be related to the nature of the semiconductor at the surface of the composite film, i.e., BiVO_4_, and/or some (photo)chemical interactions between the species present in this particular photoelectrochemical system.

**Figure 6:**
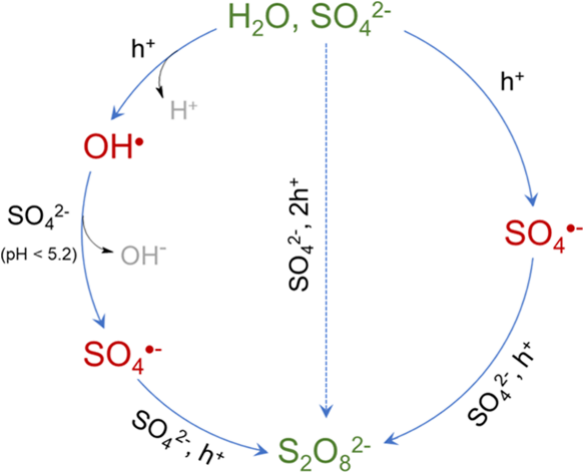
Mechanisms of persulfate ion formation in photoelectrochemical systems involving radical and nonradical pathways.

In general, it is challenging to determine the dominant role of a particular radical in the photoelectrochemical systems under investigation due to the proximity of the HO^•^/H_2_O and SO_4_^–•^/SO_4_^2–^ potentials within the working pH range (Figure 5c,d). Local variations in the pH at the photoelectrode surface can render these chemical equilibria highly dynamic. The mechanisms of radical and nonradical formation of persulfate in the photoelectrochemical systems investigated are schematically summarized in Figure 6. The higher efficiency of photoelectrochemical degradation of diclofenac found for WO_3_ could be attributed to the higher oxidizing power of VB holes, which have a larger energy offset to drive the oxidation of solution species.

### 3.5 Photoelectrode Stability Tests

**Figure 7:**
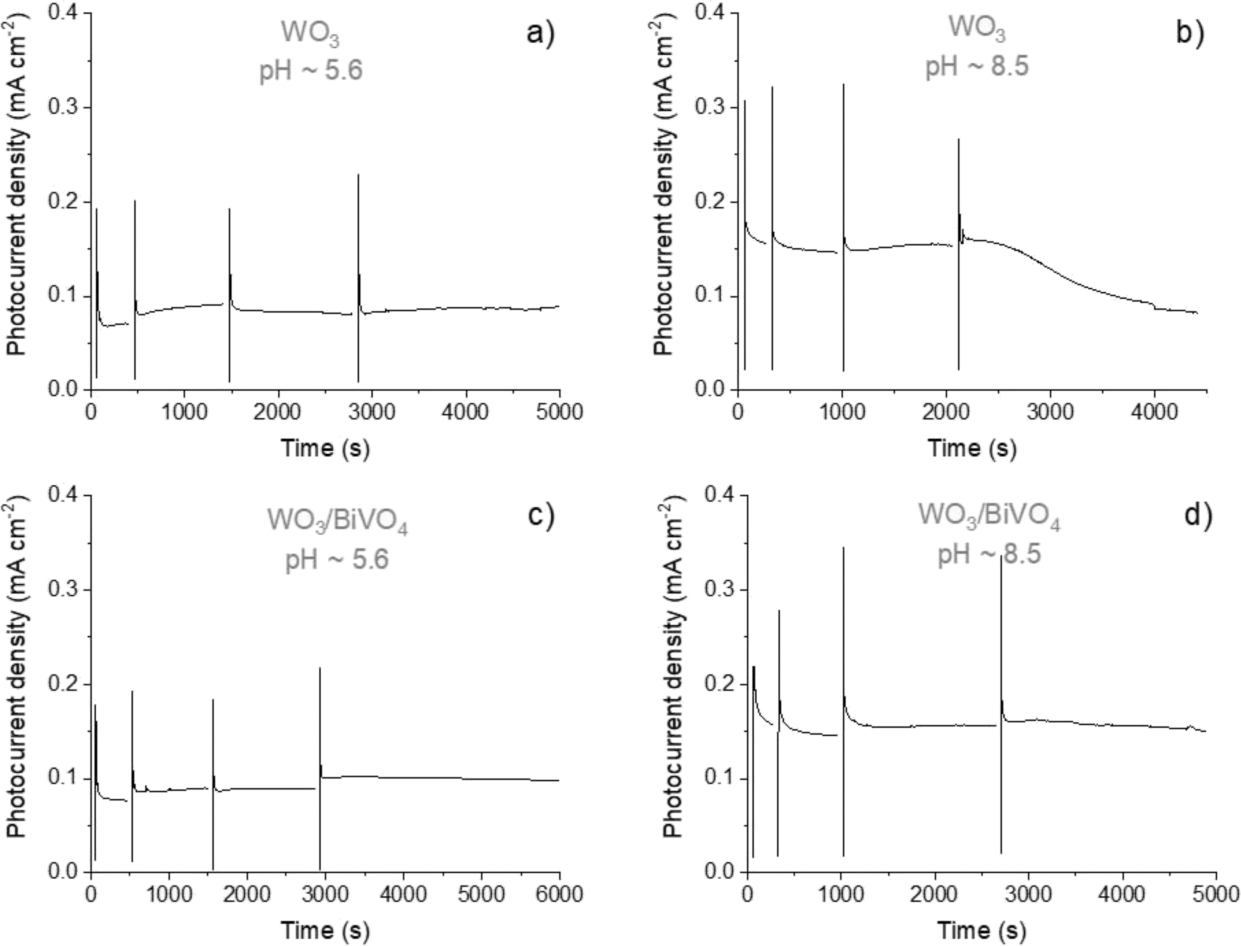
Chronoamperograms of WO_3_ (a, b) and WO_3_/BiVO_4_ (c, d) photoelectrodes recorded in unbuffered (a, c) and borate-buffered (pH = 8.5) (b, d) 0.1 M Na_2_SO_4_ electrolyte containing 50 mg L^–1^ of DCF at an applied bias of 1.4 V (a, c) and 0.8 V (a, d) (vs Pt).

WO_3_ and WO_3_/BiVO_4_ photoelectrodes were subjected to prolonged photoelectrolysis in 0.1 M Na_2_SO_4_ electrolyte containing 50 mg L^–1^ of DCF. The results of cumulative 14 h-long stability tests are presented in Figure S6. After each electrolysis session, the electrolyte was replaced with a fresh one. The results reveal satisfactory stability of photocurrent: stationary values reached within the first hour of photoelectrolysis declined by no more than 10% during the next 5–6 h in longer experiments. Another notable feature is that in each consecutive photoelectrolysis experiment, the photocurrent consistently started at nearly the same value as in the previous run, indicating partial regeneration of the photoelectrode surface. It is worth noting that a slight decrease in the pH of the electrolyte during photoelectrolysis (Figure 5c) is beneficial to the stability of WO_3_, as this semiconductor is intrinsically unstable at pH > 4.^66,67^ This was further corroborated by comparative stability tests in DCF-containing unbuffered and borate-buffered (pH 8.5) 0.1 M Na_2_SO_4_ electrolytes. A series of consecutively recorded chronoamperograms corresponding to the passage of 0.1, 0.3, 0.5, and 1 C of charge (Figure 7a–d) demonstrate stable photocurrents in all cases except for WO_3_ in borate-buffered solution with pH = 8.5. After ∼2500 s, the photocurrent started to decline, and the dissolution of the photoactive layer could be visually observed, as shown in the photos of the photoelectrodes taken before and after the experiment (Figure S7). These results indicate that heterostructuring with BiVO_4_ can be an effective strategy to protect WO_3_ from photocorrosion in solutions with pH > 4.

## 4 Conclusions

In this study, an advanced oxidation process based on the photoelectrochemical in situ generation of persulfate using WO_3_ and WO_3_/BiVO_4_ photoanodes in a simple aqueous sulfate solution under visible light illumination was demonstrated. The Faradaic efficiencies of light-induced generation of S_2_O_8_^2–^ were as high as 84 and 71% for WO_3_ and WO_3_/BiVO_4_, respectively. The decomposition of diclofenac, amoxicillin, and tetracycline was investigated, and the highest degradation efficiency of ∼80% was found for DCF. The degradation of antibiotics (AMX and TTC) occurred at a much lower rate, with efficiencies of 10–14 and 19–21%, respectively. The mechanism of light-induced degradation of DCF was investigated by comparing the results obtained under photoelectrochemical, photocatalytic, electrochemical, and chemical oxidation treatments. Decomposition of DCF under chemical treatment with S_2_O_8_^2–^ was found to be more than twice less effective compared to PEC treatment. It was suggested that persulfate in the investigated PEC systems could be generated through either nonradical or radical mechanisms. The involvement of radicals was evidenced by quenching experiments using scavengers. On the basis of the results obtained, direct (eqs i and iv) or HO^•^-mediated (eqs ii–iv) pathways of S_2_O_8_^2–^ formation were proposed.
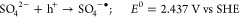
i
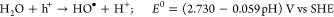
ii
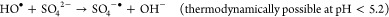
iii
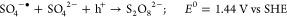
iv

The pH of the 0.1 M Na_2_SO_4_ solution during photoelectrolysis was found to decrease from 5.6 to 4.2 for both photoanodes, implying that both radical-mediated routes are possible because of the proximity of potentials of HO^•^ and SO_4_^–•^ formation under the experimental conditions of this study.

The formation of the WO_3_/BiVO_4_ heterojunction was found to improve the charge carrier separation. However, pure WO_3_ demonstrated greater effectiveness in the photoelectrochemical degradation of pharmaceutical compounds. This superior performance was attributed to the higher oxidizing potential of photogenerated holes in WO_3_, highlighting that the intrinsic properties of the semiconductor in direct contact with the electrolyte play a crucial role in determining the efficiency of the PEC degradation processes. On the other hand, the deposition of a BiVO_4_ overlayer was shown to mitigate the intrinsic instability of WO_3_ in solutions with pH > 4. The results highlight the importance of the PEC–AOS system optimization to maximize the degradation efficiency of pharmaceuticals.

## Supporting Information Available

Schematics of the photoelectrolysis cell (Figure S1); EDX spectra and cross-sectional SEM images of WO_3_ and WO_3_/BiVO_4_ samples (Figure S2); high-resolution XPS spectra of WO_3_ and WO_3_/BiVO_4_ surfaces (Figure S3); results of the evolution of the decomposition of pharmaceuticals over time in a 0.1 M Na_2_SO_4_ electrolyte that has been subjected to photoelectrolysis (Figure S4); variation of *C*/*C*_0_ of pharmaceuticals under photoelectrochemical, electrochemical, photocatalytic, and chemical oxidative treatments in 0.1 M Na_2_SO_4_ (Figure S6); and photos of WO_3_ and WO_3_/BiVO_4_ photoelectrodes before and after photoelectrolysis in unbuffered and then borate-buffered sulfate electrolytes containing DCF (PDF)

## Abbreviations

ABPE: applied bias photon-to-current efficiency
AMX: amoxicillin
AO: ammonium oxalate
AOP: advanced oxidation process
AOS: advanced oxidation systems
CA: chronoamperometry, chronoamperogram
CV: cyclic voltammetry, cyclic voltammogram
DCF: diclofenac
EDX: energy-dispersive X-ray analysis
EIS: electrochemical impedance spectroscopy
FE: Faradaic efficiency
GIXRD: grazing incidence X-ray diffraction
MeOH: methanol
PEG: poly(ethylene glycol)
PEC–AOS: photoelectrochemical advanced oxidation system
RSS: reactive sulfate species
SEM: scanning electron microscopy
SHE: standard hydrogen electrode
TBA: *tert*-butyl alcohol
TCC: tetracycline
XRD: X-ray diffraction

## References

[ref1] Morin-Crini N.; Lichtfouse E.; Fourmentin M.; Ribeiro A. R. L.; Noutsopoulos C.; Mapelli F.; Fenyvesi É.; Vieira M. G. A.; Picos-Corrales L. A.; Moreno-Piraján J. C.; Giraldo L.; Sohajda T.; Huq M. M.; Soltan J.; Torri G.; Magureanu M.; Bradu C.; Crini G. Removal of Emerging Contaminants from Wastewater Using Advanced Treatments. A Review. Environ. Chem. Lett. 2022; Vol.20, pp1333–1375.

[ref2] Phoon B. L.; Ong C. C.; Mohamed Saheed M. S.; Show P. L.; Chang J. S.; Ling T. C.; Lam S. S.; Juan J. C. Conventional and Emerging Technologies for Removal of Antibiotics from Wastewater. J. Hazard. Mater. 2020, 400, 122961 10.1016/j.jhazmat.2020.122961. 32947727

[ref3] Eniola J. O.; Kumar R.; Barakat M. A.; Rashid J. A Review on Conventional and Advanced Hybrid Technologies for Pharmaceutical Wastewater Treatment. J. Cleaner Prod. 2022, 356, 131826 10.1016/j.jclepro.2022.131826.

[ref4] Reis R. Y. N.; Goulart L. A.; Mascaro L. H.; Alves S. A. A Critical View of the Contributions of Photoelectrochemical Technology to Pharmaceutical Degradation. J. Environ. Chem. Eng. 2022, 10 (3), 107859 10.1016/j.jece.2022.107859.

[ref5] Lee J.; Von Gunten U.; Kim J. H. Persulfate-Based Advanced Oxidation: Critical Assessment of Opportunities and Roadblocks. Environ. Sci. Technol. 2020, 54 (6), 3064–3081. 10.1021/acs.est.9b07082. 32062964

[ref6] Orimolade B. O.; Arotiba O. A. Bismuth Vanadate in Photoelectrocatalytic Water Treatment Systems for the Degradation of Organics: A Review on Recent Trends. J. Electroanal. Chem. 2020, 878, 114724 10.1016/j.jelechem.2020.114724.

[ref7] Koiki B. A.; Muzenda C.; Jayeola K. D.; Zhou M.; Marken F.; Arotiba O. A. Sulfate Radical in (Photo)Electrochemical Advanced Oxidation Processes for Water Treatment: A Versatile Approach. J. Phys. Chem. Lett. 2023, 14 (39), 8880–8889. 10.1021/acs.jpclett.3c01361. 37766606 PMC10561262

[ref8] Koiki B. A.; Orimolade B. O.; Zwane B. N.; Nkwachukwu O. V.; Muzenda C.; Ojo B. O.; Nkosi D.; Mabuba N.; Arotiba O. A. Sulphate Radical Enhanced Photoelectrochemical Degradation of Sulfamethoxazole on a Fluorine Doped Tin Oxide - Copper(I) Oxide Photoanode. J. Electroanal. Chem. 2021, 900, 115714 10.1016/j.jelechem.2021.115714.

[ref9] Wacławek S.; Lutze H. V.; Grübel K.; Padil V. V. T.; Černík M.; Dionysiou D. D. Chemistry of Persulfates in Water and Wastewater Treatment: A Review. Chem. Eng. J. 2017, 330, 44–62. 10.1016/j.cej.2017.07.132.

[ref10] Liu Y.; Zhang Y.; Guo H.; Cheng X.; Liu H.; Tang W. Persulfate-Assisted Photodegradation of Diethylstilbestrol Using Monoclinic BiVO4 under Visible-Light Irradiation. Environ. Sci. Pollut. Res. 2017, 24 (4), 3739–3747. 10.1007/s11356-016-8020-3. 27888484

[ref11] Cao V. D.; Nong L. X.; Nguyen V. H.; Tran T. V.; Vu H. T.; Nguyen C. V.; Do S. T. High Degradation of BIVO4 Nanoparticle for Organic Dyes under Visible Light Irradiation Mediated by S_2_O_8_. IOP Conf. Ser. Mater. Sci. Eng. 2020, 736 (4), 042019 10.1088/1757-899X/736/4/042019.

[ref12] Murgolo S.; De Ceglie C.; Di Iaconi C.; Mascolo G. Novel TiO_2_-Based Catalysts Employed in Photocatalysis and Photoelectrocatalysis for Effective Degradation of Pharmaceuticals (PhACs) in Water: A Short Review. Curr. Opin. Green Sustainable Chem. 2021, 30, 100473 10.1016/j.cogsc.2021.100473.

[ref13] Torres-Pinto A.; Díez A. M.; Silva C. G.; Faria J. L.; Sanromán M. Á.; Silva A. M. T.; Pazos M. Photoelectrocatalytic Degradation of Pharmaceuticals Promoted by a Metal-Free G-C_3_N_4_ Catalyst. Chem. Eng. J. 2023, 476, 146761 10.1016/j.cej.2023.146761.

[ref14] Petruleviciene M.; Parvin M.; Savickaja I.; Gece G.; Naujokaitis A.; Pakstas V.; Pilipavicius J.; Gegeckas A.; Gaigalas G.; Juodkazyte J. WO_3_ Coatings for Photoelectrochemical Synthesis of Persulfate: Efficiency, Stability and Applicability. J. Solid State Electrochem. 2022, 26 (4), 1021–1035. 10.1007/s10008-022-05144-8.

[ref15] Sayama K. Production of High-Value-Added Chemicals on Oxide Semiconductor Photoanodes under Visible Light for Solar Chemical-Conversion Processes. ACS Energy Lett. 2018, 3 (5), 1093–1101. 10.1021/acsenergylett.8b00318.

[ref16] Liu X.; Gu S.; Zhao Y.; Zhou G.; Li W. BiVO4, Bi2WO6 and Bi2MoO6 Photocatalysis: A Brief Review. J. Mater. Sci. Technol. 2020, 56, 45–68. 10.1016/j.jmst.2020.04.023.

[ref17] Murillo-Sierra J. C.; Hernández-Ramírez A.; Hinojosa-Reyes L.; Guzmán-Mar J. L. A Review on the Development of Visible Light-Responsive WO_3_-Based Photocatalysts for Environmental Applications. Chem. Eng. J. Adv. 2021, 5, 100070 10.1016/j.ceja.2020.100070.

[ref18] Petruleviciene M.; Juodkazyte J.; Parvin M.; Tereshchenko A.; Ramanavicius S.; Karpicz R.; Samukaite-Bubniene U.; Ramanavicius A. Tuning the Photo-Luminescence Properties of Wo3 Layers by the Adjustment of Layer Formation Conditions. Materials 2020, 13 (12), 2814 10.3390/ma13122814. 32585794 PMC7344486

[ref19] Speldrich S.; Wark M.; Wittstock G. Metal Oxide Protection Layers for Enhanced Stability and Activity of WO3 Photoanodes in Alkaline Media. ACS Appl. Energy Mater. 2023, 6 (18), 9602–9614. 10.1021/acsaem.3c01642.

[ref20] Ta C. X. M.; Furusho Y.; Amano F. Photoelectrochemical Stability of WO_3_/Mo-Doped BiVO_4_ Heterojunctions on Different Conductive Substrates in Acidic and Neutral Media. Appl. Surf. Sci. 2021, 548, 149251 10.1016/j.apsusc.2021.149251.

[ref21] Liu J.; Li B.; Kong L.; Xiao Q.; Huang S. Surfactants-Assisted Morphological Regulation of BiVO4 Nanostructures for Photocatalytic Degradation of Organic Pollutants in Wastewater. J. Phys. Chem. Solids 2023, 172, 111079 10.1016/j.jpcs.2022.111079.

[ref22] Nguyen T. D.; Nguyen V. H.; Nanda S.; Vo D. V. N.; Nguyen V. H.; Van Tran T.; Nong L. X.; Nguyen T. T.; Bach L. G.; Abdullah B.; Hong S. S.; Van Nguyen T. BiVO4 Photocatalysis Design and Applications to Oxygen Production and Degradation of Organic Compounds: A Review. Environ. Chem. Lett. 2020, 18 (6), 1779–1801. 10.1007/s10311-020-01039-0.

[ref23] Ramanavičius S.; Petrulevičiene M.; Juodkazyte J.; Grigucevičiene A.; Ramanavičius A. Selectivity of Tungsten Oxide Synthesized by Sol-Gel Method towards Some Volatile Organic Compounds and Gaseous Materials in a Broad Range of Temperatures. Materials 2020, 13 (3), 523 10.3390/ma13030523. 31978986 PMC7040576

[ref24] Wang C.; Zhao Y.; Cheng C.; Li Q.; Guo C.; Hu Y. S-Scheme Heterojunction Photocatalysts: Mechanism, Challenges and Opportunities. Coord. Chem. Rev. 2024, 521, 216177 10.1016/j.ccr.2024.216177.

[ref25] Su J.; Guo L.; Bao N.; Grimes C. A. Nanostructured WO_3_/BiVO_4_ Heterojunction Films for Efficient Photoelectrochemical Water Splitting. Nano Lett. 2011, 11 (5), 1928–1933. 10.1021/nl2000743. 21513345

[ref26] Cheng C.; Xu H.; Ni M.; Guo C.; Zhao Y.; Hu Y. Interfacial Electron Interactions Governed Photoactivity and Selectivity Evolution of Carbon Dioxide Photoreduction with Spinel Cobalt Oxide Based Hollow Hetero-Nanocubes. Appl. Catal., B 2024, 345, 123705 10.1016/j.apcatb.2024.123705.

[ref27] Orimolade B. O.; Arotiba O. A. Enhanced Photoelectrocatalytic Degradation of Diclofenac Sodium Using a System of Ag-BiVO_4_/BiOI Anode and Ag-BiOI Cathode. Sci. Rep. 2022, 12 (1), 4214 10.1038/s41598-022-08213-0. 35273333 PMC8913733

[ref28] Sun J.; Guo Y.; Wang Y.; Cao D.; Tian S.; Xiao K.; Mao R.; Zhao X. H2O2 Assisted Photoelectrocatalytic Degradation of Diclofenac Sodium at G-C_3_N_4_/BiVO_4_ Photoanode under Visible Light Irradiation. Chem. Eng. J. 2018, 332, 312–320. 10.1016/j.cej.2017.09.041.

[ref29] Hua Z.; Dai Z.; Bai X.; Ye Z.; Wang P.; Gu H.; Huang X. Copper Nanoparticles Sensitized TiO_2_ Nanotube Arrays Electrode with Enhanced Photoelectrocatalytic Activity for Diclofenac Degradation. Chem. Eng. J. 2016, 283, 514–523. 10.1016/j.cej.2015.07.072.

[ref30] Omrani N.; Nezamzadeh-Ejhieh A. A Comprehensive Study on the Mechanism Pathways and Scavenging Agents in the Photocatalytic Activity of BiVO_4_/WO_3_ Nano-Composite. J. Water Process Eng. 2020, 33, 101094 10.1016/j.jwpe.2019.101094.

[ref31] Chi Z.; Zhao J.; Zhang Y.; Yu H.; Yu H. Coral-like WO_3_/BiVO_4_ Photoanode Constructed via Morphology and Facet Engineering for Antibiotic Wastewater Detoxification and Hydrogen Recovery. Chem. Eng. J. 2022, 428, 131817 10.1016/j.cej.2021.131817.

[ref32] Du H.; Pu W.; Wang Y.; Yan K.; Feng J.; Zhang J.; Yang C.; Gong J. Synthesis of BiVO_4_/WO_3_ Composite Film for Highly Efficient Visible Light Induced Photoelectrocatalytic Oxidation of Norfloxacin. J. Alloys Compd. 2019, 787, 284–294. 10.1016/j.jallcom.2019.01.390.

[ref33] Davies K. R.; Allan M. G.; Nagarajan S.; Townsend R.; Dunlop T.; McGettrick J. D.; Asokan V. S.; Ananthraj S.; Watson T.; Godfrey A. R.; Durrant J. R.; Maroto-Valer M. M.; Kuehnel M. F.; Pitchaimuthu S. Solar Light-Driven Simultaneous Pharmaceutical Pollutant Degradation and Green Hydrogen Production Using a Mesoporous Nanoscale WO3/BiVO4 Heterostructure Photoanode. J. Environ. Chem. Eng. 2023, 11 (3), 110256 10.1016/j.jece.2023.110256.

[ref34] Peleyeju M. G.; Viljoen E. L. WO_3_-Based Catalysts for Photocatalytic and Photoelectrocatalytic Removal of Organic Pollutants from Water – A Review. J. Water Process Eng. 2021, 40, 101930 10.1016/j.jwpe.2021.101930.

[ref35] Zeng Q.; Lyu L.; Gao Y.; Chang S.; Hu C. A Self-Sustaining Monolithic Photoelectrocatalytic/Photovoltaic System Based on a WO_3_/BiVO_4_ Photoanode and Si PVC for Efficiently Producing Clean Energy from Refractory Organics Degradation. Appl. Catal., B 2018, 238, 309–317. 10.1016/j.apcatb.2018.07.005.

[ref36] Petruleviciene M.; Turuta K.; Savickaja I.; Juodkazyte J.; Ramanavicius A. Photoelectrochemical Degradation of Organic Compounds via Formed Reactive Chlorine and Sulfate Species by WO_3_-Based Photoanodes. J. Electroanal. Chem. 2023, 951, 117954 10.1016/j.jelechem.2023.117954.

[ref37] Malefane M. E.; Feleni U.; Kuvarega A. T. Cobalt (II/III) Oxide and Tungsten (VI) Oxide p-n Heterojunction Photocatalyst for Photodegradation of Diclofenac Sodium under Visible Light. J. Environ. Chem. Eng. 2020, 8 (2), 103560 10.1016/j.jece.2019.103560.

[ref38] Pobozy E.; Kaczmarek S.; Miecznikowski K.; Pyrzynska K.; Biesaga M. Photocatalytic Degradation of Selected Non-Opioid Analgesics Driven by Solar Light Exposure. Appl. Sci. 2024, 14 (17), 7768 10.3390/app14177768.

[ref39] Mugunthan E.; Saidutta M. B.; Jagadeeshbabu P. E. Photocatalytic Activity of ZnO-WO_3_ for Diclofenac Degradation under Visible Light Irradiation. J. Photochem. Photobiol., A 2019, 383, 111993 10.1016/j.jphotochem.2019.111993.

[ref40] Cheng X.; Wang P.; Liu H. Visible-Light-Driven Photoelectrocatalytic Degradation of Diclofenac by N, S-TiO_2_/TiO_2_ NTs Photoelectrode: Performance and Mechanism Study. J. Environ. Chem. Eng. 2015, 3 (3), 1713–1719. 10.1016/j.jece.2015.06.015.

[ref41] Cheng X.; Liu H.; Chen Q.; Li J.; Wang P. Enhanced Photoelectrocatalytic Performance for Degradation of Diclofenac and Mechanism with TiO2 Nano-Particles Decorated TiO_2_ Nano-Tubes Arrays Photoelectrode. Electrochim. Acta 2013, 108, 203–210. 10.1016/j.electacta.2013.06.110.

[ref42] G B.; Banat F.; Abu Haija M. Photoelectrochemical Advanced Oxidation Processes for Simultaneous Removal of Antibiotics and Heavy Metal Ions in Wastewater Using 2D-on-2D WS_2_@CoFe_2_O_4_ Heteronanostructures. Environ. Pollut. 2023, 339, 122753 10.1016/j.envpol.2023.122753. 37852314

[ref43] Haddadou N.; Bensemma N.; Rekhila G.; Trari M.; Taïbi K. Photoelectrochemical Properties of the Relaxor Ba(Ti_0.90_Sc_0.05_Nb_0.05_)O_3_: Application to the Degradation of Amoxicillin under Solar Light. J. Mater. Sci.: Mater. Electron. 2018, 29 (6), 5042–5048. 10.1007/s10854-017-8466-1.

[ref44] Wang J.; Jiang L.; Liu F.; Jia M.; Liu M.; Li J.; Lai Y. Enhanced Photoelectrochemical Degradation of Tetracycline Hydrochloride with FeOOH and Au Nanoparticles Decorated WO_3_. Chem. Eng. J. 2021, 407, 127195 10.1016/j.cej.2020.127195.

[ref45] Le H. V.; Le L. T.; Han H.; Ung T. T. D.; Tran P. D. Photoelectrodeposition of Ag_3_PO_4_ Nanoparticles on BiVO_4_ Photoanode for Enhancing Its Photoelectrochemical Water Oxidation Performance. J. Sci. Adv. Mater. Devices 2023, 8 (2), 100547 10.1016/j.jsamd.2023.100547.

[ref46] Chen Z.; N Dinh H.; Miller E. Photoelectrochemical Water Splitting Standards, Experimental Methods, and Protocols; Springer: New York, NY, 2013.

[ref47] Armstrong D. A.; Huie R. E.; Koppenol W. H.; Lymar S. V.; Merenyi G.; Neta P.; Ruscic B.; Stanbury D. M.; Steenken S.; Wardman P. Standard Electrode Potentials Involving Radicals in Aqueous Solution: Inorganic Radicals (IUPAC Technical Report). Pure Appl. Chem. 2015, 87 (11–12), 1139–1150. 10.1515/pac-2014-0502.

[ref48] Parvin M.; Petrulevičienė M.; Savickaja I.; Šebeka B.; Karpicz R.; Grigucevičienė A.; Ramanauskas R.; Juodkazytė J. Influence of Morphology on Photoanodic Behaviour of WO_3_ Films in Chloride and Sulphate Electrolytes. Electrochim. Acta 2022, 403, 139710 10.1016/j.electacta.2021.139710.

[ref49] Szilágyi I. M.; Janos M.; Pokol G.; Kiraly P.; Gabor T.; Sami S.; Janos M.; Attila T.; Andras S.; Katalin V.-J. Stability and Controlled Composition of Hexagonal WO_3_. Chem. Mater. 2008, 20 (12), 4116–4125. 10.1021/cm800668x.

[ref50] Wei P.; Wen Y.; Lin K.; Li X. 2D/3D WO_3_/BiVO_4_ Heterostructures for Efficient Photoelectrocatalytic Water Splitting. Int. J. Hydrogen Energy 2021, 46 (54), 27506–27515. 10.1016/j.ijhydene.2021.06.007.

[ref51] Gutkowski R.; Khare C.; Conzuelo F.; Kayran Y. U.; Ludwig A.; Schuhmann W. Unraveling Compositional Effects on the Light-Induced Oxygen Evolution in Bi(V-Mo-X)O4Material Libraries. Energy Environ. Sci. 2017, 10 (5), 1213–1221. 10.1039/C7EE00287D.

[ref52] Wu J. M.; Chen Y.; Pan L.; Wang P.; Cui Y.; Kong D. C.; Wang L.; Zhang X.; Zou J. J. Multi-Layer Monoclinic BiVO_4_ with Oxygen Vacancies and V^4+^ Species for Highly Efficient Visible-Light Photoelectrochemical Applications. Appl. Catal., B 2018, 221, 187–195. 10.1016/j.apcatb.2017.09.031.

[ref53] Shi X.; Wu Q.; Cui C. Modulating WO_3_ Crystal Orientation to Suppress Hydroxyl Radicals for Sustainable Solar Water Oxidation. ACS Catal. 2023, 13 (2), 1470–1476. 10.1021/acscatal.2c05325.

[ref54] Cristino V.; Pasti L.; Marchetti N.; Berardi S.; Bignozzi C. A.; Molinari A.; Passabi F.; Caramori S.; Amidani L.; Orlandi M.; Bazzanella N.; Piccioni A.; Kopula Kesavan J.; Boscherini F.; Pasquini L. Photoelectrocatalytic Degradation of Emerging Contaminants at WO_3_/BiVO_4_ Photoanodes in Aqueous Solution. Photochem. Photobiol. Sci. 2019, 18 (9), 2150–2163. 10.1039/c9pp00043g. 30931455

[ref55] Wang J.; Wang S. Reactive Species in Advanced Oxidation Processes: Formation, Identification and Reaction Mechanism. Chem. Eng. J. 2020, 401, 126158 10.1016/j.cej.2020.126158.

[ref56] Kumari M.; Pulimi M. Sulfate Radical-Based Degradation of Organic Pollutants: A Review on Application of Metal-Organic Frameworks as Catalysts. ACS Omega 2023, 8 (38), 34262–34280. 10.1021/acsomega.3c02977. 37779959 PMC10536895

[ref57] Monfort O.; Plesch G. Bismuth Vanadate-Based Semiconductor Photocatalysts: A Short Critical Review on the Efficiency and the Mechanism of Photodegradation of Organic Pollutants. Environ. Sci. Pollut. Res. 2018, 25 (20), 19362–19379. 10.1007/s11356-018-2437-9. 29860700

[ref58] Sun B.; Li H.; Wei Q.; Xue S.; Zhou A.; Yue X. Enhanced Quinoline Degradation by Persulfate-Assisted Photocatalytic Process with WO_3_-CuFe_2_O_4_ Z-Scheme System: Properties and Mechanism. Sep. Purif. Technol. 2022, 301, 122039 10.1016/j.seppur.2022.122039.

[ref59] Ambrosio F.; Wiktor J.; Pasquarello A. PH-Dependent Catalytic Reaction Pathway for Water Splitting at the BiVO_4_-Water Interface from the Band Alignment. ACS Energy Lett. 2018, 3 (4), 829–834. 10.1021/acsenergylett.8b00104.

[ref60] Chen S.; Wang L. W. Thermodynamic Oxidation and Reduction Potentials of Photocatalytic Semiconductors in Aqueous Solution. Chem. Mater. 2012, 24 (18), 3659–3666. 10.1021/cm302533s.

[ref61] Bacha A. U. R.; Nabi I.; Cheng H.; Li K.; Ajmal S.; Wang T.; Zhang L. Photoelectrocatalytic Degradation of Endocrine-Disruptor Bisphenol – A with Significantly Activated Peroxymonosulfate by Co-BiVO_4_ Photoanode. Chem. Eng. J. 2020, 389, 124482 10.1016/j.cej.2020.124482.

[ref62] Wang L.; Liu Z.; Xu X.; Jia Y.; Mei Q.; Ding F.; Peng J.; Wang Q. Efficient Solar Water Splitting via Enhanced Charge Separation of the BiVO_4_ Photoanode. ACS Appl. Energy Mater. 2022, 5 (5), 6383–6392. 10.1021/acsaem.2c00779.

[ref63] Nasseri S.; Mahvi A. H.; Seyedsalehi M.; Yaghmaeian K.; Nabizadeh R.; Alimohammadi M.; Safari G. H. Degradation Kinetics of Tetracycline in Aqueous Solutions Using Peroxydisulfate Activated by Ultrasound Irradiation: Effect of Radical Scavenger and Water Matrix. J. Mol. Liq. 2017, 241, 704–714. 10.1016/j.molliq.2017.05.137.

[ref64] Paquin F.; Rivnay J.; Salleo A.; Stingelin N.; Silva C. Multi-Phase Semicrystalline Microstructures Drive Exciton Dissociation in Neat Plastic Semiconductors. J. Mater. Chem. C 2015, 3, 10715–10722. 10.1039/C5TC02043C.

[ref65] Liang C.; Su H. W. Identification of Sulfate and Hydroxyl Radicals in Thermally Activated Persulfate. Ind. Eng. Chem. Res. 2009, 48 (11), 5558–5562. 10.1021/ie9002848.

[ref66] Anik M. PH-Dependent Anodic Reaction Behavior of Tungsten in Acidic Phosphate Solutions. Electrochim. Acta 2009, 54 (15), 3943–3951. 10.1016/j.electacta.2009.02.014.

[ref67] Nave M. I.; Kornev K. G. Complexity of Products of Tungsten Corrosion: Comparison of the 3D Pourbaix Diagrams with the Experimental Data. Metall. Mater. Trans. A 2017, 48 (3), 1414–1424. 10.1007/s11661-016-3888-6.

