# Supplementary material for: Photoelectrochemical Degradation of Diclofenac, Tetracycline, and Amoxicillin in an Aqueous Sulfate Medium: Analysis of Reactive Species
Source: ACS Omega. 2025 Feb 17;10(8):8538–50. doi: 10.1021/acsomega.4c10891 (PMC11886689; doi:10.1021/acsomega.4c10891)
Supplement: Supplementary file 1 — ao4c10891_si_001.pdf [file ao4c10891_si_001.pdf]

Photoelectrochemical degradation of diclofenac, tetracycline, and amoxicillin in aqueous sulfate medium: analysis of reactive species

Milda Petruleviciene[1], Irena Savickaja[1], Jelena Kovger-Jarosevic[1], Jurga Juodkazyte[1], Audrius Padarauskas[2], Asta Griguceviciene[1] and Arunas Ramanavicius[3,4]*

[1]Centre for Physical Sciences and Technology, Department of Chemical Engineering and Technology, Sauletekio av. 3, LT-10257, Vilnius, Lithuania;

[2]Institute of Chemistry, Faculty of Chemistry and Geosciences, Department of Analytical and Environmental Chemistry, Vilnius University, Naugarduko 24, LT- 03225, Vilnius, Lithuania

[3]Institute of Chemistry, Faculty of Chemistry and Geosciences, Department of Physical Chemistry, Vilnius University, Naugarduko 24, LT- 03225, Vilnius, Lithuania

[4] Centre for Physical Sciences and Technology, Department of Nanotechnology, Sauletekio av. 3, LT-10257, Vilnius, Lithuania;

Supporting information

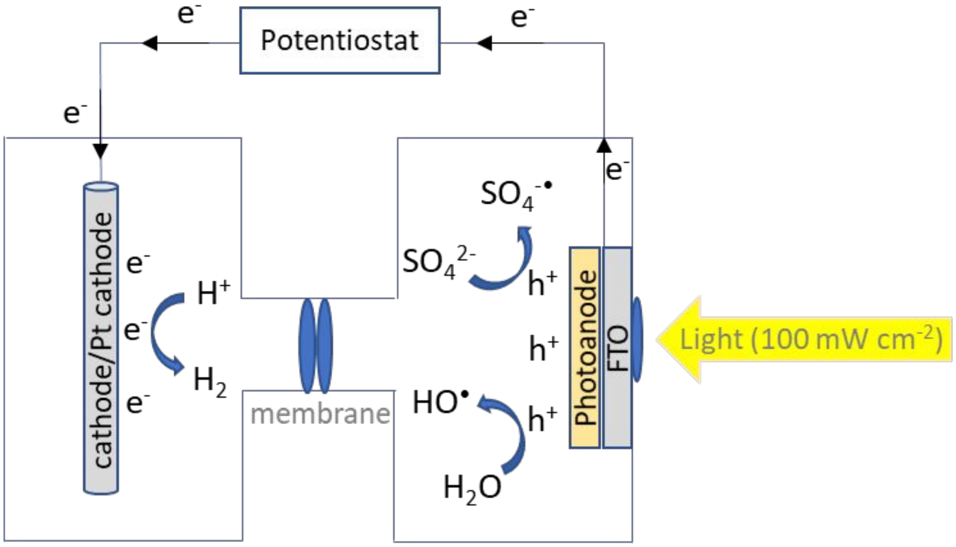

Figure S1. Schematic view of H-type electrochemical cell used for photoelectrolysis

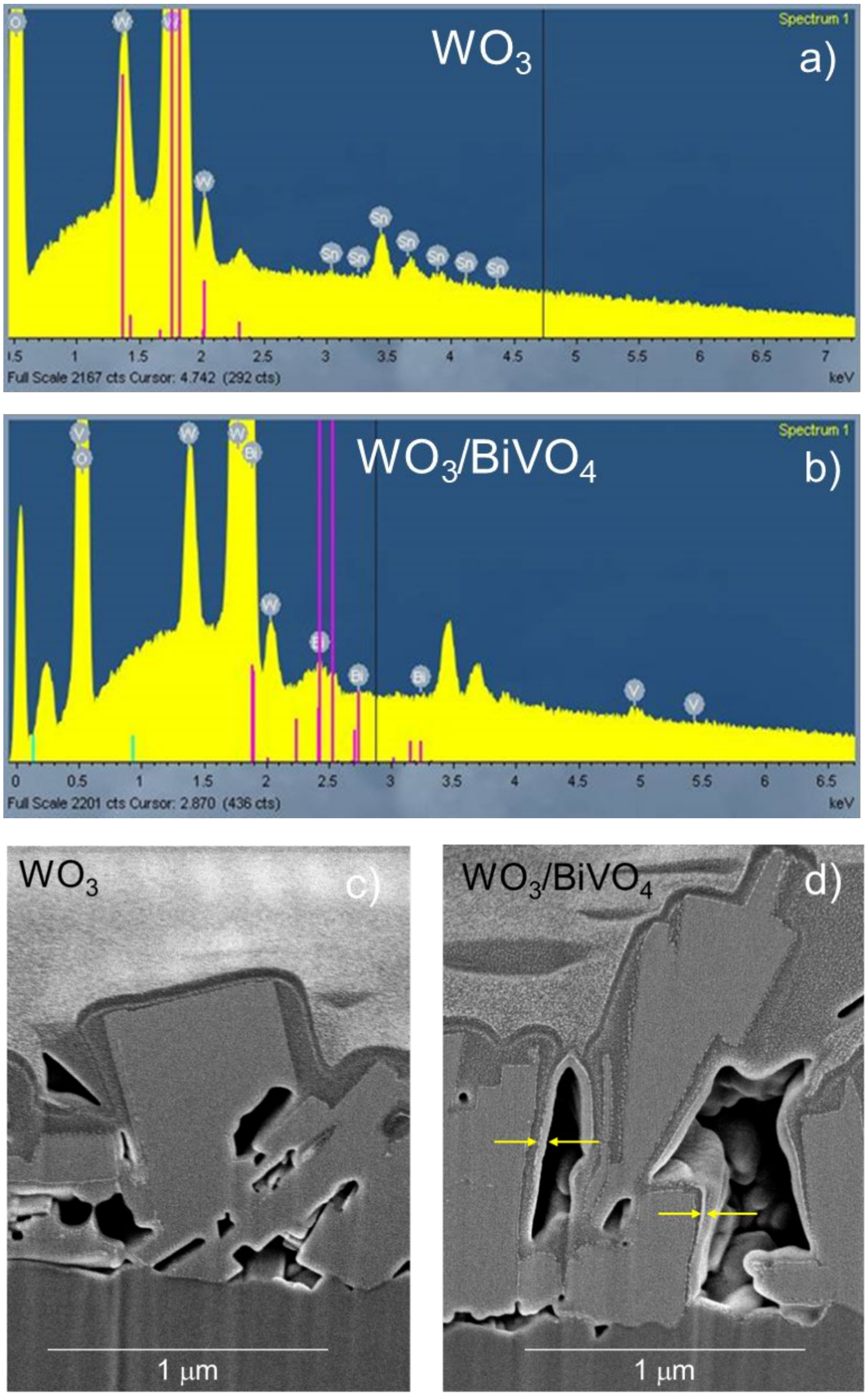

Figure S2. EDX spectra (a, b) and cross-sectional SEM images (c, d) of WO_3 (a, c) and $WO_3/BiVO_4$ (b, d) coatings.

Peaks corresponding to Bi and V elements are observed in EDX spectra. Yellow arrows in SEM image (d) denote thin (10-30 nm) $BiVO_4$ coating on WO_3

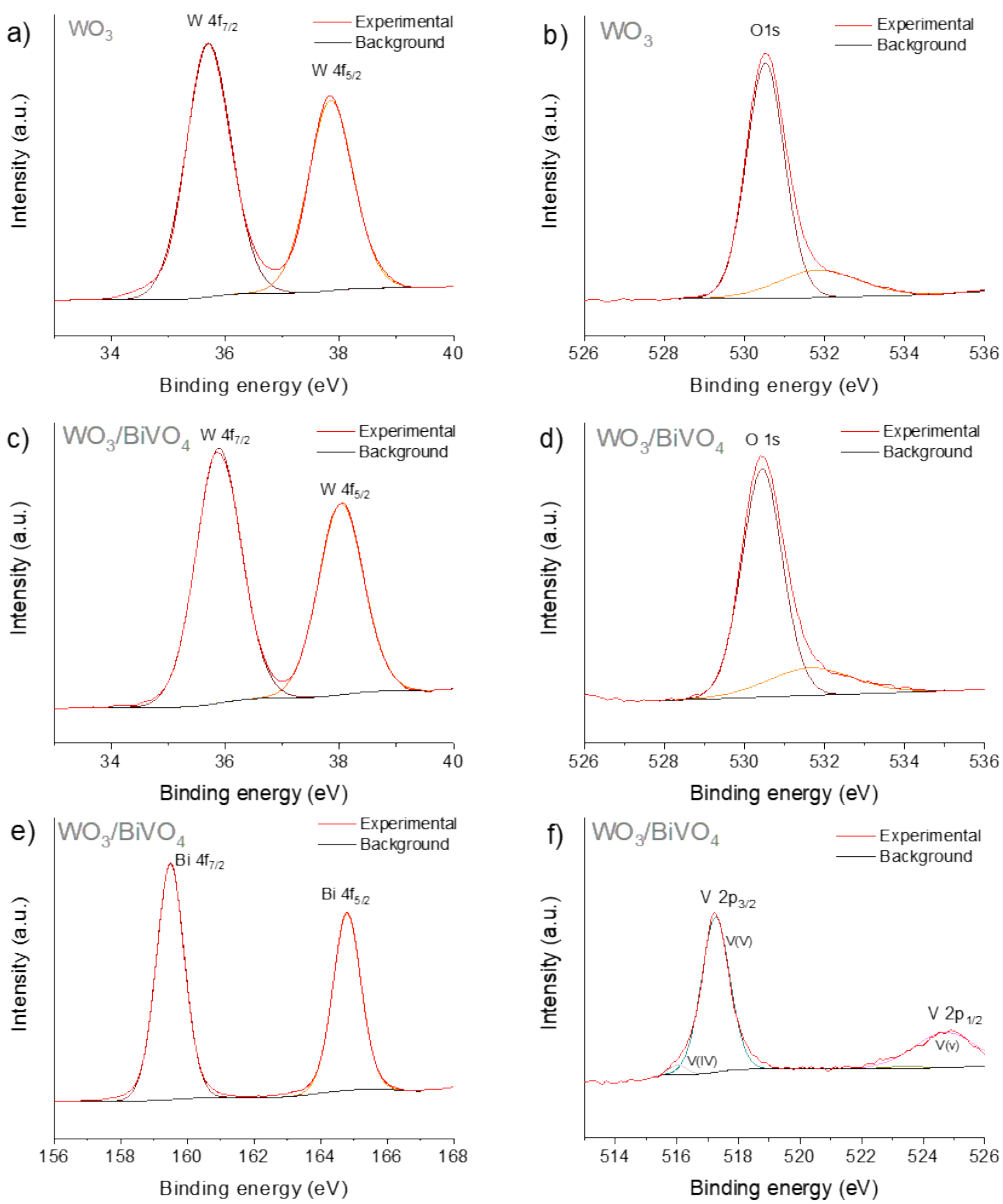

Figure S3. XPS core spectra of (a, c) W4f and (b, d) O1s recorded for WO_3 (a, b) and $WO_3/BiVO_4$ (c, d). XPS core spectra of (e) Bi4f and (f) V2p measured from $WO_3/BiVO_4$. The red line represents experimental data, and solid yellow, burgundy, green, grey and bluish lines denote the data fitting by CASA software.

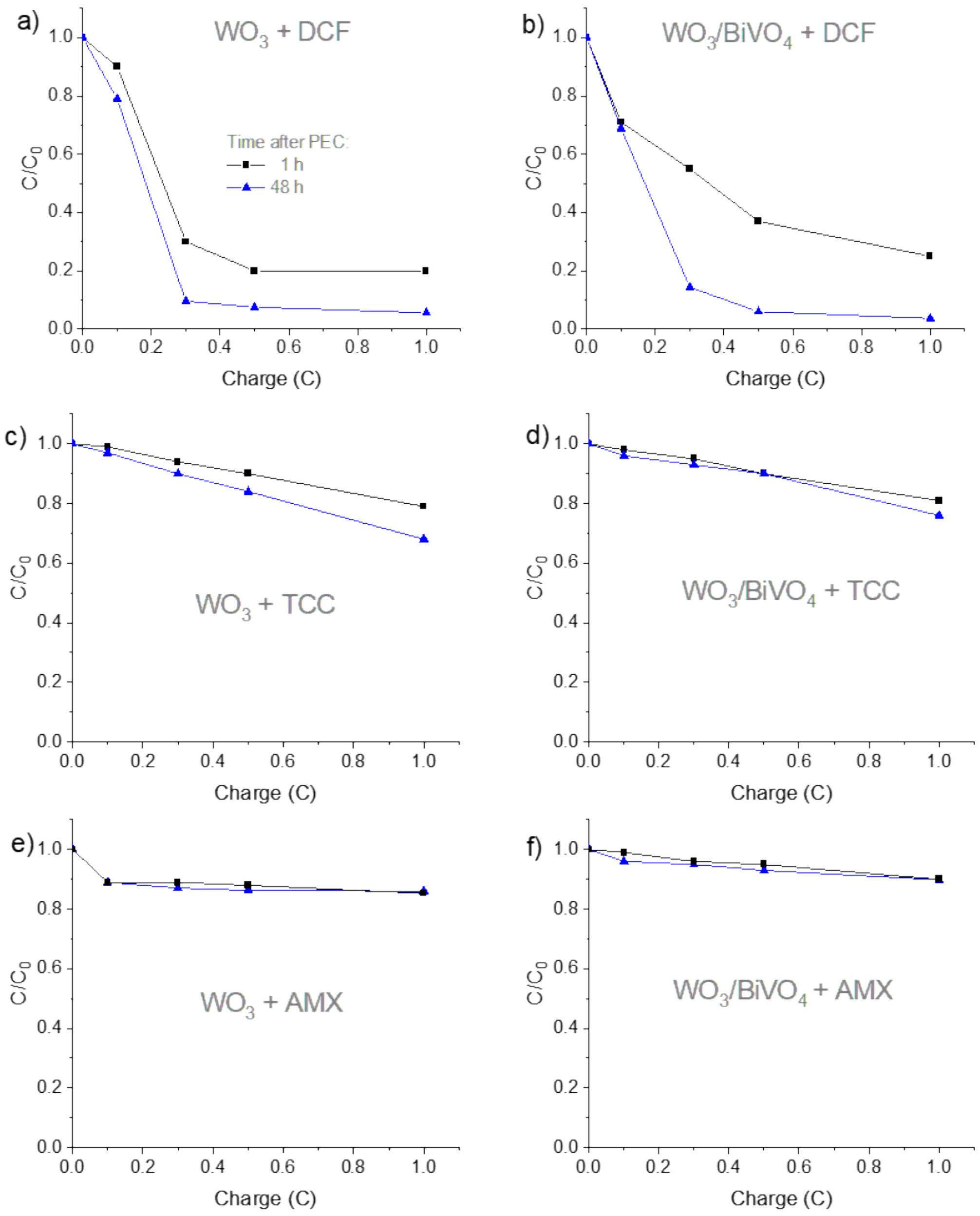

Figure S4. Progression of the decomposition of DCF (a, b), AMX (c, d), and TCC (e, f) with time in 0.1 M Na_2SO_4 electrolyte subjected to PEC treatment with WO_3 (a, d, e) or $WO_3/BiVO_4$ (b, d, f) photoanodes.

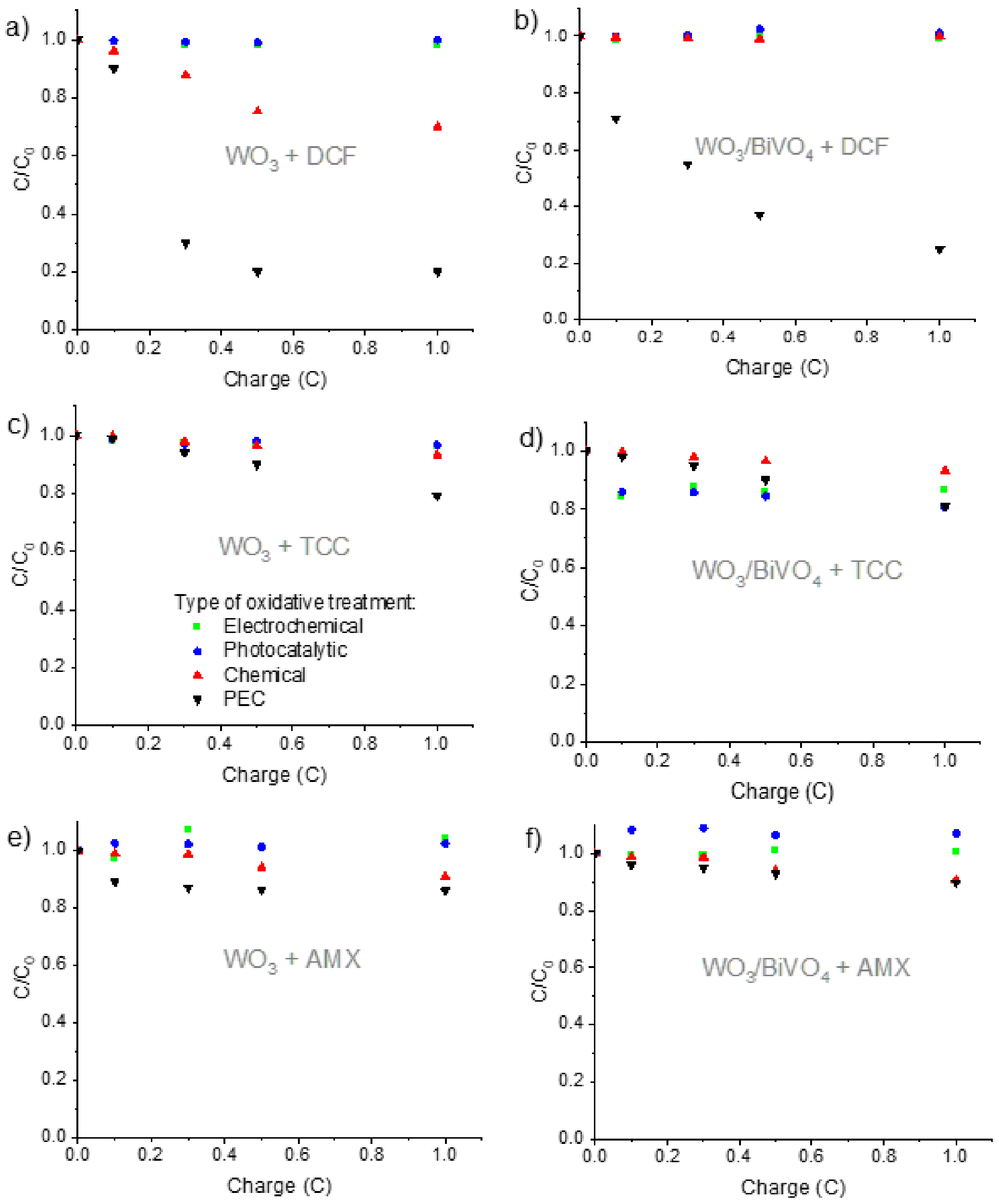

Figure. S5. Variation of normalized concentration ratio of DFC (a, b), TTC (c, d) and AMX (e, f) under different oxidative treatment conditions with WO_3 (a, c, e) and $WO_3/BiVO_4$ (b, d, f) photoanodes. See Experimental section for details.

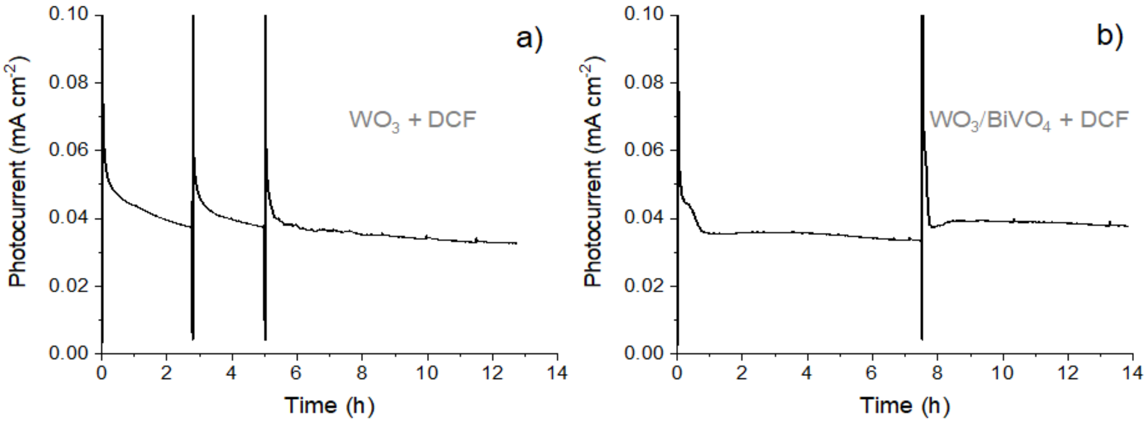

Figure S6. Cumulative stability tests of WO_3 (a) and $WO_3/BiVO_4$ (b) photoanodes during consecutive photoelectrolysis sessions in 0.1 M Na_2SO_4 + 50 mg L^{-1} DCF at 1.4 V (*vs* Pt); after each, session the electrolyte was replaced with a fresh one

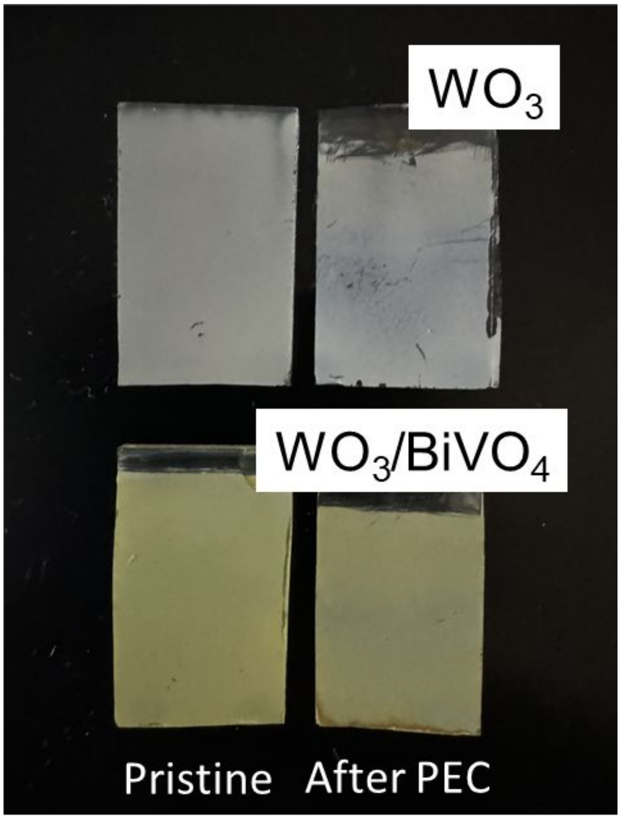

Figure S7. Photos of WO_3 and $WO_3/BiVO_4$ photoanodes before and after photoelectrolysis performed sequentially in unbuffered and then borate-buffered (pH = 8.5) 0.1 M Na_2SO_4 electrolyte containing 50 mg L^{-1} of DCF (see Figure 7).
